# Lipidomic comparison of 2D and 3D colon cancer cell culture models

**DOI:** 10.1002/jms.4880

**Published:** 2022-08-26

**Authors:** Fernando Tobias, Amanda B. Hummon

**Affiliations:** ^1^ Department of Chemistry and Biochemistry The Ohio State University Columbus Ohio USA; ^2^ Comprehensive Cancer Center The Ohio State University Columbus Ohio USA

**Keywords:** acidosis, cancer, fatty acid/metabolism, hypoxia, lipid droplets, mass spectrometry, serial trypsinization, spheroids, TME, triacylglycerol

## Abstract

Altered lipid metabolism is one of the hallmarks of cancer. Cellular proliferation and de novo synthesis of lipids are related to cancer progression. In this study, we evaluated the lipidomic profile of two‐dimensional (2D) monolayer and multicellular tumor spheroids from the HCT 116 colon carcinoma cell line. We utilized serial trypsinization on the spheroid samples to generate three cellular populations representing the proliferative, quiescent, and necrotic regions of the spheroid. This analysis enabled a comprehensive identification and quantification of lipids produced in each of the spheroid layer and 2D cultures. We show that lipid subclasses associated with lipid droplets form in oxygen‐restricted and acidic regions of spheroids and are produced at higher levels than in 2D cultures. Additionally, sphingolipid production, which is implicated in cell death and survival pathways, is higher in spheroids relative to 2D cells. Finally, we show that increased numbers of lipids composed of polyunsaturated fatty acids (PUFAs) are produced in the quiescent and necrotic regions of the spheroid. The lipidomic signature for each region and cell culture type highlights the importance of understanding the spatial aspects of cancer biology. These results provide additional lipid biomarkers in colon cancer cells that can be further studied to target pivotal lipid production pathways.

## 1 INTRODUCTION

Cancer is a major global public health problem and is the second leading cause of death in the United States.^1^ Colorectal cancer is projected to be the third most diagnosed cancer, and the third leading cause of cancer‐related death in the United States for 2022.^2^ Cancer cells are characterized by their increased proliferation, resistance to apoptosis, and poorly differentiated character. To better understand the disease, human cell line models have been essential tools to study these characteristics for in vitro research. Although two‐dimensional (2D) cell cultures, where the cells are grown in monolayers or in suspension, are most commonly used, there are limitations to this configuration. Namely, cells grown in 2D lack the complexity and number of cell–cell connections that occur in tissues.

Three‐dimensional (3D) cell cultures enable substantial improvements in mimicking the tumor microenvironment and recapitulating other critical aspects of tumor biology better than traditional monolayer (2D) culturing platforms. Although 2D cell cultures have minimal cell–cell contacts and a homogenous phenotype, the multicellular tumor spheroid (MCTS) is an example of a 3D cell culture that provides a platform for examining cellular function and metabolism in cancer.^3^, ^4^ Nutrient and oxygen concentration gradients are formed in spheroids, much like tumors, and consist of proliferating cells in the outer layers and hypoxic, nutrient‐deprived necrotic cells in the inner regions.^5^, ^6^ In between these vastly different cell populations, viable quiescent cells are also present.^6^

Studies on the altered lipid metabolism of colorectal cancers show it to be a promising and targetable vulnerability.^7^ Mass spectrometry‐based lipidomic studies on colorectal cancer have taken a number of approaches to understand lipidomic alterations better as they relate to different cell lines, the effect of co‐culturing with different cell types, or comparing patient tumor and adjacent normal tissues. For example, Rombouts et al. conducted lipidomics and a polar metabolomics profiling among different colorectal cancer cell lines at different cancer stages and normal colon cell lines.^8^ Metabolites involved in lipid synthesis were shown to be elevated in colon cancer cell lines. Additionally, they found a correlation between increased overall phosphatidylcholine (PC) lipid content and metastatic potential, especially between HT‐29 and HCT 116, in which HT‐29 cells have greater metastatic potential and have a higher PC content.

Studies involving cancer therapeutics suggest specific lipid profile changes after treatment. These lipid profile alterations are often unique based on the cell type and the therapeutic administered, which suggests that lipid metabolism remodeling is very complex in colorectal cancer. For example, it was revealed that phosphoglycerolipid levels increased, and triacylglycerol levels decreased in HT‐29 cells after oxaliplatin treatment.^9^ Meanwhile, Jung et al. investigated and quantified the effect of 5‐fluorouracil (5‐FU) on different colorectal cancer cell lines that were sensitive and resistant to the drug therapy, in which they found altered levels of sphingolipids, such as sphingomyelins (SM) and ceramides (Cer).^10^ Several SM species were significantly up‐regulated in 5‐FU‐resistant samples, whereas Cer species were significantly down‐regulated. Sphingolipids are bioactive molecules that have critical roles in regulating cancer cells. SMs have pro‐survival function whereas Cers can mediate cell death.^11^ The use of copper oxide nanoparticles has also been investigated to induce toxicity in HCT 116 cells, where a dose‐dependent increase of certain ceramide species was observed, as well as the upregulation of triacylglycerols, and phosphatidylcholines.^12^

Co‐culturing cells have increasingly been recognized as a technique to understand cell‐to‐cell communication and to better model the tumor microenvironment.^13^ Gong et al. investigated the role of cancer‐associated fibroblasts in modifying the lipidome after co‐culturing with DLD‐1 cells. This study suggests that lipidomic reprogramming and increased tumor metastasis is due to lipid metabolite crosstalk between cell types.^14^

Studies involving patient tumor samples and matched nondiseased mucosa samples can provide the most significant clinical relevance. To date, several studies have reported lipidomic alterations in patient samples, but there are large variations between individual studies.^15^, ^16^ Recently, Ecker et al. reported a lipidomics study conducted on patient tumor samples diagnosed with primary colorectal cancer.^17^ Using three independent patient cohorts, they have identified lipidomic changes in tumor samples compared with normal tissue. Despite a high degree of variation among the cohorts, which the authors suggested was due to patients' diet, prescribed drugs and other confounding metabolic disorders, significant differences in glycerolipids, glycerophospholipids, and sphingolipids were identified. They found certain sphingomyelin (SM) and triacylglycerol (TG) species to be elevated and proposed a lipid signature based on TG levels which differentiated them from nontumor tissue. This study provided a comprehensive analysis of CRC patient samples and suggested putative markers to diagnose patients better and to monitor potential drug therapies.

One of the critical aspects of using spheroids as a tumor model is that their cellular composition is similar to an in vivo tumor. As spheroids grow, radially symmetric chemical gradients develop. Many of the current approaches in generating tumor spheroids can be produced at a large scale, generate uniform structures, and be used for cancer‐related studies with live‐cell imaging,^18^ immunohistochemistry, Western blotting, mass spectrometry‐based proteomics,^19^, ^20^ and metabolomics.^21^, ^22^, ^23^ Recent drug toxicity studies of cancer therapeutics were conducted using mass spectrometry imaging (MSI) of spheroids, which provides the spatial distribution of not only the drug therapeutic but also drug metabolites.^24^, ^25^ This label‐free imaging technology can also be used to profile other biochemical changes, such as small molecule metabolites involved in the Krebs cycle and lipids.^26^, ^27^, ^28^ Within colorectal cancer research, MSI has been previously used to characterize lipid profiles of tumor tissues and the adjacent environment.^29^

To mitigate the loss of spatial information, serial trypsinization can be subsequently utilized after harvesting spheroids. Using a dilute trypsin solution, cells can be sequentially peeled off from the spheroid through multiple cycles and washes. McMahon et al. previously obtained discrete cell populations from HT‐29 spheroids representing different regions of the 3D culture by subjecting them to serial trypsin treatments, quantifying proteins in HT‐29 spheroids involved in several cellular metabolism pathways.^30^ Keithley et al. also utilized serial trypsinization to probe the sphingolipid metabolic profile at different regions of HCT 116 spheroids. They were able to detect fluorescent probes that were specifically labeled for the outer, middle, and core regions after serial trypsinization in a reproducible manner and showed cells from different regions exhibited differences in metabolism.^31^

In this work, we conducted comprehensive lipidomics profiling of the HCT 116 colon carcinoma cell line as 3D MCTS and 2D monolayers. For each biological replicate, we conducted serial trypsinization to further elucidate the lipidomic changes in different regions of MCTS before lipid extraction. Liquid chromatography coupled to high‐resolution quadrupole time‐of‐flight mass spectrometry was utilized to identify and quantify lipids. We found several classes of lipids that are highly altered in spheroids compared with 2D monolayer cultures. The lipidomic profile of the other regions shows similar features as 2D monolayers, whereas the middle (quiescent) and core (necrotic) regions diverge from this similarity and show unique lipidomic features that suggest altered lipid metabolism and different energy storage. Triacylglycerols and certain classes of sphingolipids were elevated in the hypoxic and necrotic regions of HCT 116 spheroids, suggesting the 3D microenvironment of spheroids affects the lipid spatial distribution. Further, lipids consisting of fatty acyls with longer carbon‐chain and a higher degree of fatty acyl unsaturation are observed in spheroids at higher levels relative to 2D monolayers. Finally, we discuss our lipidomic results in the context of recent studies on the effects hypoxic and low‐pH regions of the tumor microenvironment.

## 2 MATERIALS AND METHODS

### 2.1 Chemicals

Methyl *tert*‐butyl ether (MTBE) and isopropanol (LiChrosolv‐grade) were obtained from EMD Millipore (Sigma‐Millipore, St. Louis, Missouri, USA). Methanol and water (LC–MS grade) were obtained from Honeywell, acetonitrile (Optima LC–MS grade) was obtained from Fisher Scientific (Charlotte, North Carolina, USA), and solid ammonium formate (LC–MS LiChropur‐grade) was obtained from Sigma‐Millipore (St. Louis, Missouri, USA).

### 2.2 Spheroid culturing

The colon carcinoma cell line HCT 116 was purchased from the American Type Culture Collection (ATCC, Manassas, Virginia, USA). It was cultured as a two‐dimensional (2D) monolayer in McCoy's 5A cell culture media (Life Technologies, Grand Island, New York, USA), supplemented with 10% fetal bovine serum (FBS) (Invitrogen, San Diego, California, USA) and 1% l‐glutamine. The cells were placed in T25 flask and grown in 5% CO_2_ at 37°C until confluency.

Spheroids were cultured in agarose‐coated 96‐well plates as previously described.^32^, ^33^ Briefly, the outer wells of every 96‐well plate had 200 μl 1× PBS to prevent evaporation of cell culture media; 65 μl agarose, dissolved in McCoy's 5A without FBS supplementation, was placed in the 60 inner wells of the 96‐well plates. Cell suspensions from the 2D monolayer cultures were seeded in each well at 7000 cells per well using an 8‐channel pipettor. The spheroids were grown in 5% CO_2_ at 37°C, and 50% of the culture media was changed every 48 h after 4 days of growth. After 14 days of growth, spheroids were rinsed with 1× PBS, before being transferred into Petri dishes to be subjected through serial trypsinization.

### 2.3 Spheroid embedding, cryosectioning, and Oil Red O staining

A separate 96‐well plate of spheroids were cultured for Oil Red O staining. At Day 14, spheroids were transferred into one well of a 12‐well plate, where the spheroids were washed with 1× PBS to remove residual culture medium. An 81‐well 3D Petri Dish mold by Microtissues, Inc. (Sigma‐Millipore, St. Louis, Missouri, USA) was used to make a gelatin array. As discussed by Johnson et al., the molds provide better alignment of the spheroids along the *z*‐axis of the gelatin mold so multiple spheroids can be cryosectioned together.^34^ Briefly, gelatin powder was dissolved in water and warmed in a pressure cooker that is set to “warm” mode. To form the gelatin base using the mold, 550 μl of warm gelatin was carefully pipetted into the mold and allow it to harden. Using a wide‐bore pipette, spheroids were transferred with minimal 1× PBS. Residual PBS was removed around the spheroids to avoid deterioration during the cryosectioning process. Small drops of warm gelatin were placed onto each individual spheroid to embed them in place. The mold was then placed in a −80°C freezer for 10 min to allow it to harden. The spheroids were capped by carefully pipetting 190 μl of warm gelatin, and the sample was stored in a −80°C freezer until the cryosectioning step.

Cryosectioning was performed on a Leica CM 1950 cryostat (Leica Biosystems, Buffalo Grove, Illinois, USA). Spheroids were sectioned into 12 μm slices and thaw‐mounted onto glass slides. Glass slides were then stored in a −80°C freezer until the staining procedure was conducted.

Frozen glass slides were defrosted under vacuum conditions using a vacuum centrifuge chamber. A stock solution of Oil Red O was created by dissolving 300 mg of the powder in 100 ml isopropanol. Coplin jars were used to treat the slide for the following steps: 10% neutral buffered formalin for 10 min, quickly dipped in 60% isopropanol, stained in an Oil Red O solution (30 ml stock solution mixed with 20 ml water) for 15 min, quickly dipped in 60% isopropanol, counterstained with Mayer's hematoxylin (Sigma‐Millipore, St. Louis, Missouri, USA) for 3 min, dipped in deionized water 10 times, and then finally coverslipped with SlowFade Diamond Antifade Mountant (Invitrogen, Waltham, Massachusetts, USA). Stained spheroid sections were viewed using an ImageXpress Pico Automated Cell Imaging System (Molecular Devices, San Jose, California, USA) using their calorimetry mode.

### 2.4 Serial trypsinization

HyClone trypsin, 0.05% (GE Life Technologies) was used. Serial trypsinization protocol was followed as previously described.^35^

### 2.5 Lipid extraction

Serial trypsinized samples were resuspended 1× PBS and were subjected to probe sonication. A Pierce bicinchoninic acid (BCA) Protein Assay Kit (Thermo Scientific, Rockford, Illinois, USA) was used to estimate the sample protein content. In 2.0 ml microcentrifuge tubes, 10 μl of equiSPLASH LIPIDOMIX internal standards (Avanti Polar Lipids, Alabaster, Alabama, USA) were added, and aliquots containing 100–200 μg protein were subjected to lipid extraction using a modified protocol by Matyash et al.^36^ Briefly, 300 μl (150 μl for samples containing 100 μg protein) of cold methanol was added to the tube and was vortexed for 10 s; 1000 μl (500 μl) of cold MTBE was added and further vortexed for 10 s and sonicated using a bath sonicator for 5 min. Phase separation was induced by adding 250 μl (125 μl) MS‐grade water and then further vortexed for 20 s and sonicated for 5 min. The samples were centrifuged at 14,000 rpm for 2 min. The upper organic phase of each sample was collected into a new 2.0 ml microcentrifuge tube using a Hamilton syringe, rinsing between samples using MTBE and water. An additional 300 μl (150 μl) of MTBE was further added to the first set of tubes containing the lower aqueous layer. The samples were centrifuged again at 14,000 rpm for 2 min, and the upper organic layer was combined into the new 2.0 ml tube. The samples were evaporated using a vacuum centrifuge and were stored at −80°C until mass spectrometry analysis.

### 2.6 Liquid chromatography‐mass spectrometry

Dried lipid extracts were resuspended in the appropriate volume of 9:1 methanol/toluene solution to obtain an equivalent protein concentration of 2 μg/μl per sample. A quality control (QC) sample was subsequently generated which was composed of equi‐volumes of each sample. This QC sample was analyzed in Auto‐MSMS mode, whereas each biological sample was analyzed in MS1‐only. The QC sample was injected after every eighth injection and at regular intervals. Samples were injected on a 1260 Infinity HPLC (Agilent Technologies Inc., Santa Clara, California, USA) onto an Accucore C30 column (2.6 μm, 100 × 2.1 mm) (Thermo Scientific, Waltham, Massachusetts, USA) for reverse‐phase separation which was heated at 50°C with a constant flow rate at 0.400 ml/min, using a gradient of mobile phase A (50:50, acetonitrile/water, 10 mM ammonium formate) and mobile phase B (90:10 isopropanol/acetonitrile, 10 mM ammonium formate). The gradient program was as follows: 0–5 min, 15%–40%; 5–30 min, 40%–95% B; 30–32 min, hold at 95% B; 10 min, equilibrate at 15% B. Positive and negative ion mode acquisition were utilized on an Agilent 6545 quadrupole time‐of‐flight mass spectrometer equipped with a JetStream ionization source (Agilent Technologies Inc., Santa Clara, California, USA) to profile the lipid extracts, using 4 and 10 μg equivalent protein concentration, respectively.

### 2.7 Data analysis and statistics

Agilent (.d) data files were converted to ABF format using ABF Converter. MS‐DIAL version (4.48) was used for peak peaking, alignment, and identification.^37^, ^38^ The analysis parameter settings for MS‐DIAL are available in Table S2. Further, EQUISPLASH lipidomics internal standards were used to normalize and quantify the endogenous lipids detected and identified in the study as pmol lipid/μg protein. Based on the pre‐extraction volume of the internal standards in each sample and the injected sample volume for LC–MS analysis, the calculated concentrations of the internal standards are provided in the supporting information. The extracted ion chromatograms of lipid internal standards were manually assessed to ensure samples were loaded consistently into the LC–MS. Representative ion chromatograms can be found in the Figure S1. Extensive manual curation of the lipid table and their corresponding MS/MS reference spectra were conducted within MS‐DIAL and Microsoft Excel. Lipids identified by tandem MS spectra in the QC sample with a coefficient of variation (CV) greater than 20% were parsed out and were no longer considered for analysis. To further assess the reliability of the in silico database matches of the identified lipids, we compared the retention time behavior of each lipid class relative with their double‐bond number and total number of carbons in their fatty acyl chains. All retention time behavior plots are included in the supporting information. Furthermore, lipid nomenclature used in this study follows the rules from Liebisch et al.^39^ Lipids with a known fatty acyl composition based on its tandem‐MS data in negative ion mode were annotated at the “molecular species level.” Lipids that have uncertain fatty acyl composition were annotated at the “species level” that contains the total carbon and total double bond (or double bond equivalents) of the fatty acyl residues. Additionally, manual filtering between polarities were conducted to minimize multiple annotations of the same lipid. In silico annotation of the tandem‐MS of cholesterol was conducted using MS‐FINDER version (3.52).^40^ R‐based packages lipidR and SCOPE were utilized to obtain bioinformatics.^41^, ^42^ Lipids that were identified as having the same lipid carbon : double bond assignment were not filtered and annotated as either an “_A” or “(1)” for SCOPE and lipidR, respectively.

## 3 RESULTS

### 3.1 Untargeted lipidomics profiling of 2D monolayer cultures and serial trypsinized spheroids

**FIGURE 1:**
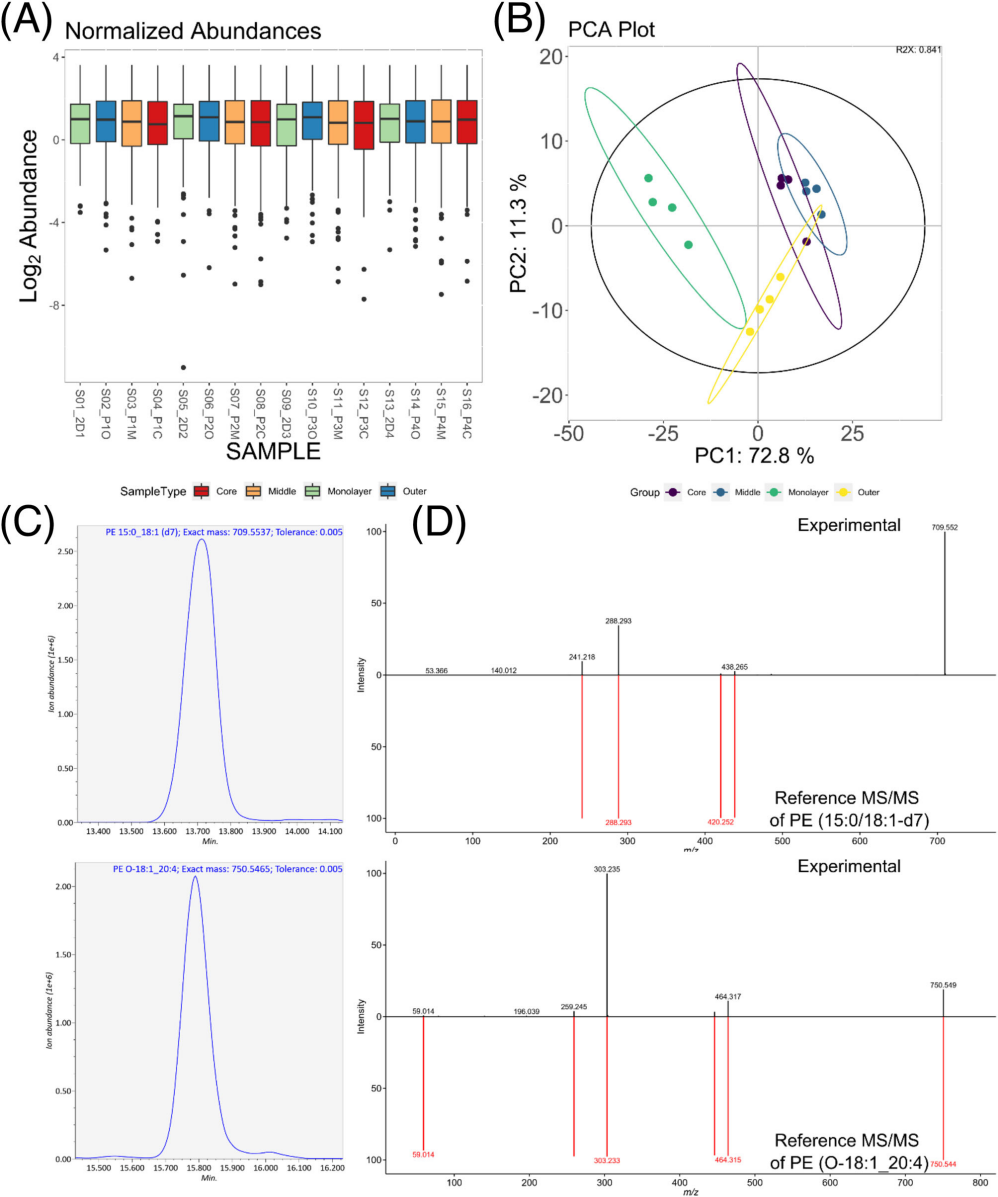
Quantitative lipidomic analysis of HCT 116 spheroids and 2D monolayer cultures. (A) Quality Control (QC) plot shows the sum of log_2_‐transformed abundance for each sample. (B) Principal Component Analysis (PCA) plot of each sample group. (C) Representative extraction chromatograms of the PE internal standard PE (15:0/18:1‐d7), and an endogenous ether‐linked phosphatidylethanolamine PE (O‐18:1_20:4). (D) MS/MS spectra assigned to the peak groups in the study representing PE internal standard PE (15:0/18:1‐d7) and PE (O‐18:1_20:4) from MS‐DIAL.

Multicellular tumor spheroids (MCTS) were generated from the colon carcinoma HCT 116 cell line to characterize and identify the lipidome in a comprehensive manner. Biological replicates were formed from pools of HCT 116 spheroids (89 spheroids per replicate) and subjected to serial trypsinization to obtain three separate cell populations, representing the outer (proliferating), middle (quiescent), and core (necrotic) regions. A representative growth curve from one 96‐well plate shows the growth throughout the 14‐day period (Figure S1). Using 2D monolayer cultures for a baseline comparison, it enabled us to conduct a lipidomic profiling of a 3D cell culture in biological quadruplicate. In total, 335 lipid species, covering four lipid categories (glycerolipids, glycerophospholipids, sphingolipids, and fatty acyls), were detected. These lipids were identified by MS/MS, which was searched against the *in‐silico* database within MS‐DIAL. An example comparison between experimental and reference tandem mass spectra and extraction ion chromatograms are illustrated in Figure 1C,D, for the internal standard PE (15:0/18:1‐d7) and an endogenous ether‐linked PE (O‐18:1_20:4). Untargeted lipidomic workflows enable quality control plots of the log_2_ transformed‐intensities which illustrate consistent levels for each sample (Figure 1A). Principal component analysis of each biological replicate shows samples was grouped by their sample type, with the 2D monolayer samples being the farthest from the origin (Figure 1B).

Based on the normalized total lipid content, the percent composition of each lipid subclass was determined between 2D monolayer and spheroid regions. As listed in Table 1, the fatty acid and PC subclasses dominated the much of the composition in the HCT 116 cell line, regardless of culturing method. In total, there were 18 lipid subclasses in this study.

**TABLE 1:** Lipid composition of HCT 116 2D monolayers and 3D spheroid regions

Subclass	2D (%mol ± SD)	Outer (%mol ± SD)	Middle (%mol ± SD)	Core (%mol ± SD)
FA	53.24 ± 2.80	56.99 ± 0.99	55.09 ± 2.83	57.88 ± 2.44
PC	30.45 ± 1.88	28.71 ± 2.07	28.91 ± 2.39	28.71 ± 1.25
Ether PC	9.79 ± 0.61	5.38 ± 0.49	8.04 ± 0.54	6.16 ± 0.61
PI	2.99 ± 0.28	2.96 ± 0.22	3.15 ± 0.26	3.14 ± 0.32
DG	1.06 ± 0.07	1.63 ± 0.60	1.23 ± 0.31	1.09 ± 0.10
Lysolipids	1.01 ± 0.31	2.24 ± 1.49	0.95 ± 0.32	0.64 ± 0.39
Ether PE	0.54 ± 0.04	0.76 ± 0.03	0.79 ± 0.05	0.78 ± 0.08
PE	0.37 ± 0.06	0.48 ± 0.05	0.43 ± 0.05	0.46 ± 0.06
TG	0.08 ± 0.01	0.28 ± 0.07	0.64 ± 0.03	0.45 ± 0.05
DMPE	0.16 ± 0.02	0.16 ± 0.01	0.17 ± 0.02	0.17 ± 0.02
SM	0.11 ± 0.01	0.13 ± 0.01	0.16 ± 0.01	0.15 ± 0.02
PS	0.13 ± 0.01	0.12 ± 0.01	0.13 ± 0.01	0.14 ± 0.01
Cer	0.03 ± 0.01	0.07 ± 0.01	0.17 ± 0.02	0.09 ± 0.02
SHexCer	0.03 ± 0.01	0.05 ± 0.004	0.07 ± 0.01	0.06 ± 0.01
CAR	4.0E‐03 ± 0.001	1.8E‐02 ± 0.01	2.6E‐02 ± 0.004	5.2E‐02 ± 0.03
HexCer	5.1E‐03 ± 0.001	1.4E‐02 ± 0.002	2.4E‐02 ± 0.004	2.3E‐02 ± 0.004
PG	4.7E‐03 ± 0.002	8.9E‐03 ± 0.001	1.2E‐02 ± 0.004	9.2E‐03 ± 0.002
NAE	3.3E‐04 ± 1.9E‐05	5.1E‐04 ± 5.2E‐05	9.5E‐04 ± 1.3E‐04	9.6E‐04 ± 9.4E‐05

### 3.2 Glycerolipids

**FIGURE 2:**
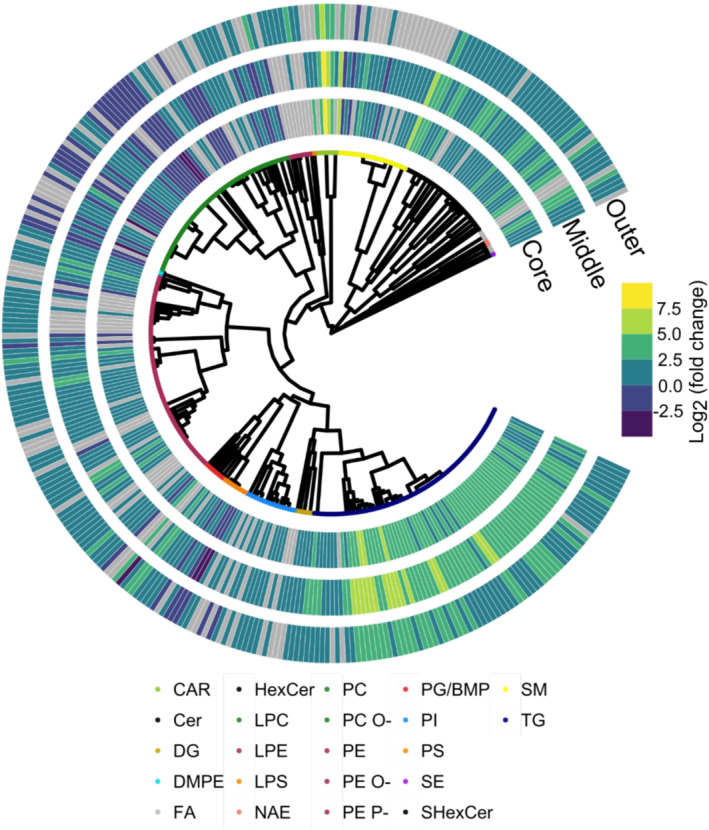
Circular dendrogram of statistically significant lipids in at least one layer using SCOPE. Lipids that have been identified at the MS/MS level are shown and clustered according to their SMILES annotation from MS‐DIAL. The lipid abundance are depicted as log_2_ fold change ratios between a spheroid layer and the 2D monolayer culture.

To provide a comprehensive spatial view of the lipid alteration within spheroids, we utilized SCOPE by Odenkirk et al. to generate a circular dendrogram that clusters lipids by their SMILES data and provides a heatmap of log_2_ fold change ratios relative to a control.^41^ This enabled a spatial comparison of each spheroid layer to further visualize the lipidomic changes at both a subclass‐wide and even on a lipid species‐specific level. Using the 2D monolayer data for our baseline comparison, Figure 2 illustrates all the lipid abundance alterations in the three spheroid regions. Based on this circular dendrogram, there are entire lipid subclasses that appear to be altered in specific spatial regions of the spheroids. For example, the triacylglycerols (TG) is an example of a subclass that are elevated in spheroids. TG are a subclass of neutral lipids that are produced by cells as either a precursor for lipid membrane synthesis, produced to sequester free fatty acids, or as a storage for energy. TGs have also been observed to accumulate in cells exposed to hypoxia due to the induction of lipin 1, as a catalyst in the triglycerol biosynthesis, and by hypoxia‐inducible transcription factors (HIFs).^43^

**FIGURE 3:**
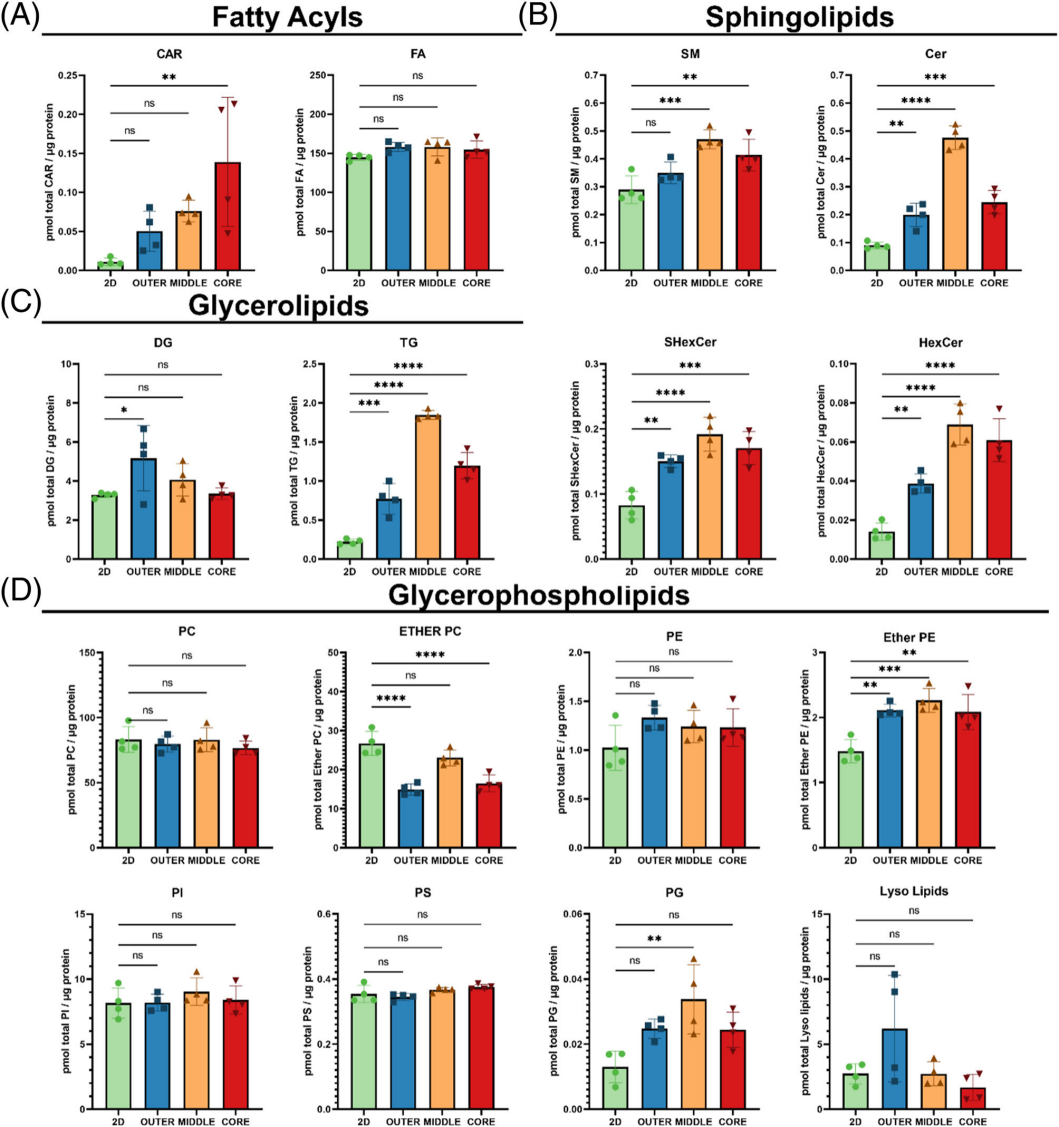
Lipid subclass abundance in 2D monolayer cultures and different regions of 3D spheroids. (A) Fatty acyls category encompasses acylcarnitines (CAR) and fatty acids (FA); (B) sphingolipids category as sphingomyelins (SM), ceramides (Cer), sulfatides (SHexCer), and hexosylceramides (HexCer); (C) glycerolipids category as diacylglycerols (DG) and triacylglycerols (TG); and (D) glycerophospholipids category as phosphatidylcholines (PC), ether‐linked PCs (PC‐O), phophatidylethanolamines (PE), ether‐linked PEs (PE‐O, PE‐P), phosphatidylinositol (PI), phosphatidylserines (PS), phosphatidylglycerol (PG), and lysophospholipids (LPC, LPE, LPI, and LPS). Statistical significance was determined using ordinary one‐way ANOVA with Bonferroni's multiple comparisons test against 2D culture samples. Adjusted *P*‐value indicators are *P < 0.05, **P < 0.005, ***P < 0.0005, ****P < 0.0001 (Figure 4).

Quantitatively, comparing the normalized molar abundance of the TG subclass further reveals the difference between 2D monolayer cultures and the spheroid regions. As illustrated in Figure 3C, there is significantly more TG content in all spheroid regions than in 2D monolayers. At the same time, diacyglycerol (DG) molar abundance remains largely remains unchanged, except for in the outer region where it is slightly elevated. To investigate the lipid species‐specific alteration for each spheroid layer, Figure 3A illustrates a heatmap as a log_2_ fold change ratio for each spheroid layer relative to the 2D culture with respect to their total carbon chain length and total chain unsaturation. The TG abundance profile emerged as a diverse lipid subclass across the three spheroid layers, which suggests different degrees of alterations. Across all layers of the spheroid, a subclass‐wide elevation of all TG species is observed, ranging from 0.2‐ to 6.2‐fold change ratios. The greatest elevation is observed for the TGs in the middle layer consisting of ≥5 double bonds and ≥50 total carbons in the fatty acyl (FA) chain. At the same time, modest elevation (0.2 to 1.2‐fold change ratios) is observed for saturated (no double bonds) TG and having <50 total carbons in the FA chain. These data may suggest that 2D cultures cannot form TG with higher degree of unsaturation.

**FIGURE 4:**
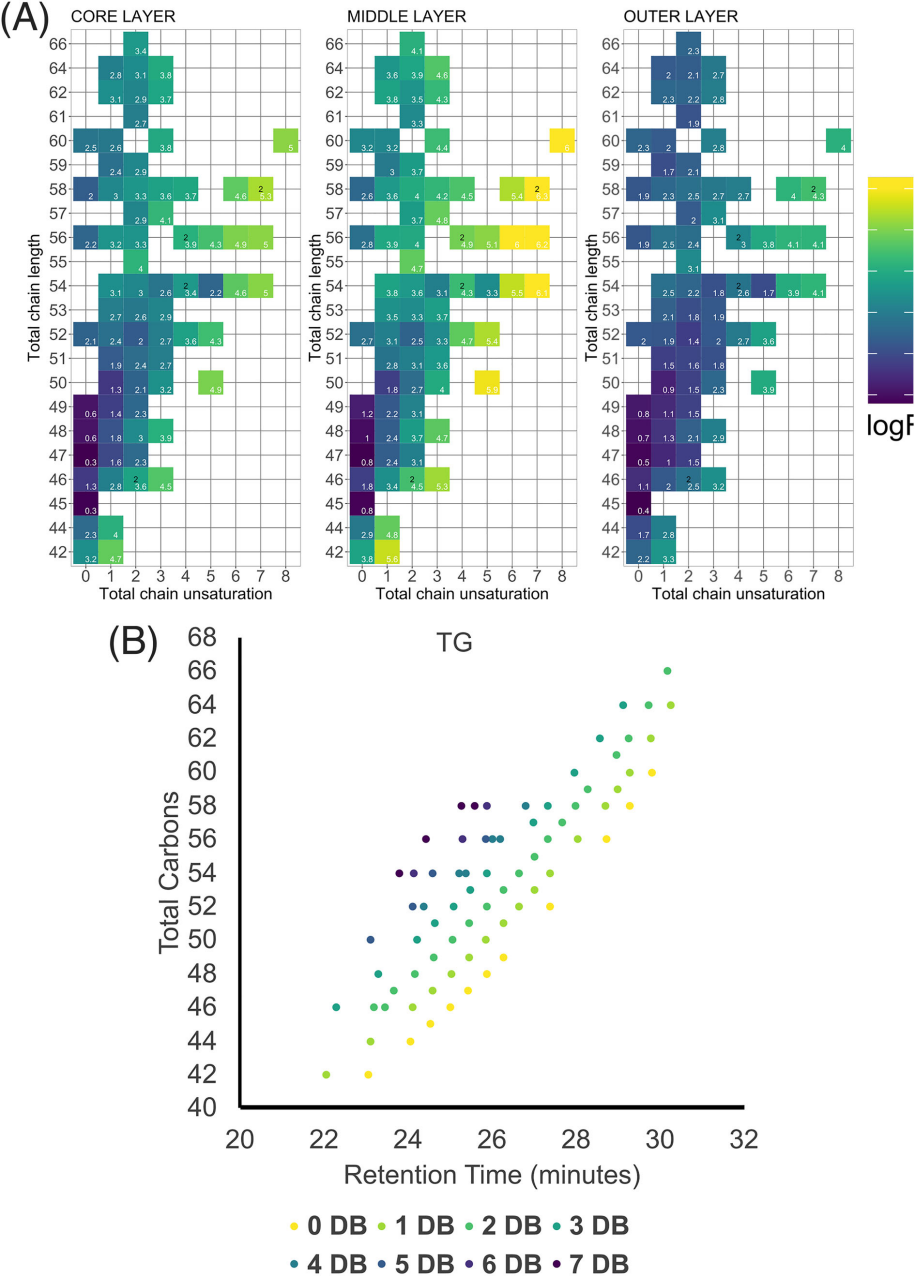
Triacylglycerol (TG) abundance profile in HCT 116 spheroids and retention time behavior of TGs in reversed‐phase chromatography. (A) Relative to monolayer cultures, higher abundance of TG lipids are present in 3D cultures that consists of higher degree of double bond unsaturation. This distinction is more amplified in the middle and core layers than in the outer layers. The numerical values inside each pixel correspond to the fold change ratio (spheroid layer/2D monolayer) of that lipid species. (B) Retention time behavior of all TG species detected and identified by MS/MS in the study; 0 dB through 7 dB represent the cumulative number of double bonds in the fatty acyl chains, whereas the total carbon represents the cumulative number of carbons in the fatty acyl chains.

Using Oil Red O staining, the nonpolar lipid distribution was observed in cryosections of spheroids (Figure S11). It is apparent in the center region of the cryosections of spheroids contain a large amount of lipid droplets (Figure S11B). Indeed, the accumulation of lipid droplets corresponds with the higher abundance of TGs in the middle and core samples in the lipidomics analysis.

### 3.3 Glycerophospholipids

Phosphatidylcholine (PC) is the most abundant glycerophospholipid that makes up much of the membranes of cells. Compared with other glycerophospholipids, PCs are present in both the outer and inner leaflets of cell membranes, as identified in a recent study by Lorent et al.^44^ The phosphatidylcholine region of Figure 2 is composed of PC and ether‐linked PC‐O species and indicates a more complex alteration profile than TG lipids. Although total PC subclass molar abundance suggests, there is no difference between 2D cultures and the spheroid regions as depicted in Figure 3D, and the total PC‐O subclass abundance suggests significant decrease in the outer and core regions of HCT 116 spheroids. On the other hand, some PC species such as PC(40:8) are highly elevated in the spheroid outer region relative to 2D cultures, as illustrated in Figure S2 with fold change ratio of 2.4. However, its molar abundances between 2D cultures and the outer spheroid region are 0.0706 and 0.3410 pmol/μg protein, respectively, which is still a fraction of the subclass total of 83.14 and 79.65 pmol/μg protein, for 2D cultures and the outer spheroid region, respectively. Meanwhile, the PC‐O heat map in Figure S2B indicates that the most altered PC lipid expression between 3D and 2D cultures are PC(O‐34:0) (core layer) and PC(O‐36:5) (middle layer), exhibiting a fold change ratio of −2.3 and 2.8, respectively.

Phosphatidylethanolamine (PE) is the second most abundant glycerophospholipid, which constitutes 20%–50% of total phospholipids.^45^ We quantified PE lipids to be third most abundant in HCT 116 2D monolayers and 3D spheroids as shown in Table 1. The phosphatidylethanolamine region of Figure 2 is composed of PE, ether‐linked (PE‐O), and plasmalogen (PE‐P) species, and a diverse panel was detected in the study and differentially expressed, with a total of 71 PE main class. The fold change expression for each PE subclass as a function of total chain length and chain unsaturation is illustrated in Figure S3A. Quantitatively, ether‐linked and plamalogen species are all significantly elevated in spheroids compared with 2D monolayer (Figure 3D). The greatest alterations appear among the PE‐O lipids where PE(O‐16:1_20:0) is highly elevated in all spatial regions.

Phosphatidylinositol (PI) is an acidic phospholipid that is normally detected in negative ion mode. Unlike PC lipids, PI lipids have been observed to be more abundant in the inner leaflet of cell membranes.^44^ A total of 18 PI lipids were detected and quantified (Figure S4). As seen in Figure 3D, there were no significant changes in the amount of PI lipids between the spheroid regions and 2D monolayer. Table S1 elaborates on the differential expression for each PI lipid. An overall higher lipid expression for every PI species in all three spheroid layers except for PI(18:0_18:1), PI(18:0_20:1), and PI(18:0_21:2). Interestingly, the most prominent alteration observed for a PI lipid between the 3D and 2D cultures was PI(16:1_16:1).

Phosphatidylserines (PS), which are mainly present in the inner leaflet of cell membranes, were detected to be differentially abundant in spheroids as compared with 2D monolayers.^44^ Nine PS lipids were identified, where two isomers identify as PS(34:2), in the current study. Across all sample types, the normalized total abundance of the PS subclass remains unchanged (Figure 3D). The major PS species detected with highest molar abundance is PS(18:0_18:1), which interestingly, had an increasing amount from the outer to the core regions (supporting information).

### 3.4 Sphingolipids

Sphingolipids are a diverse group of lipids comprising of ceramides (Cer), hexosylceramides (HexCer), sphingomyelins (SM), and sulfatides (SHexCer). In total, we detected and quantified 15 Cer, 10 HexCer, 8 ST, and 24 SM. As shown in Figure S6, many of the sphingolipid species identified were elevated in all three regions of the HCT 116 spheroids, with the exception of Cer (d34:2) (core region), SM (d33:1) (all layers), SM (d34:1) (all layers), and SM (d34:2) (all layers). Within the spheroids, higher levels of the identified Cer, HexCer, SHexCer, and SM lipids were found in the middle and core regions, which represents the quiescent and necrotic regions of the spheroid. Quantitatively, the total normalized abundance of each subclass is illustrated in Figure 3B.

### 3.5 Cholesterol, acylcarnitines, and free fatty acids

Free cholesterol was determined to not be significantly altered across spheroid regions and 2D monolayers (Figure S12B). Acylcarnitines are esters of l‐carnitine and fatty acids and are involved in the β‐oxidation of fatty acids in the mitochondria. Long‐chain acyl‐CoA synthetase (LACS) facilitates the transformation of fatty acids into acyl‐CoAs, which are then further transformed into acylcarnitines by carnitine palmitoyl‐transferase 1 and 2 (CPT1 and CPT2). Finally, acylcarnitines are transported across the mitochondrial membrane by carnitine/acylcarnitine translocase (CACT).^46^ In the current study, eight acylcarnitine species were detected and quantified across the spheroid layers and 2D cultures. As shown in Figure S7, all acylcarnitine species have relatively elevated levels across the spheroid layers as compared with 2D monolayers, although the core region has a more significant amount of CAR compared to 2D cultures (Figure 3A). CAR (22:0) is observed to have the greatest fold change among the acylcarnitine species, with a gradual increase from the outer layer to the core. The retention time behavior for the acylcarnitines is shown in Figure S9A.

Free fatty acids were also detected and quantified in this study, with seven species being detected and quantified across samples. Fatty acids comprised the majority of the lipids detected and quantified in all sample types, as shown in Table 1. A modest fold change increase (0.1–0.5) are observed in FA(16:0), FA(17:0), and FA(18:0) in the outer and middle layers. Interestingly, the most significant difference in FAs were observed in FA(24:0), having the greatest elevation in the middle layer with a fold change ratio of 2.6 (Figures S8 and S9B). Overall, the FA subspecies remains unchanged across 2D monolayers and spheroid regions (Figure 3A).

### 3.6 Chain length comparison across spheroid layers relative to 2D cultures

**FIGURE 5:**
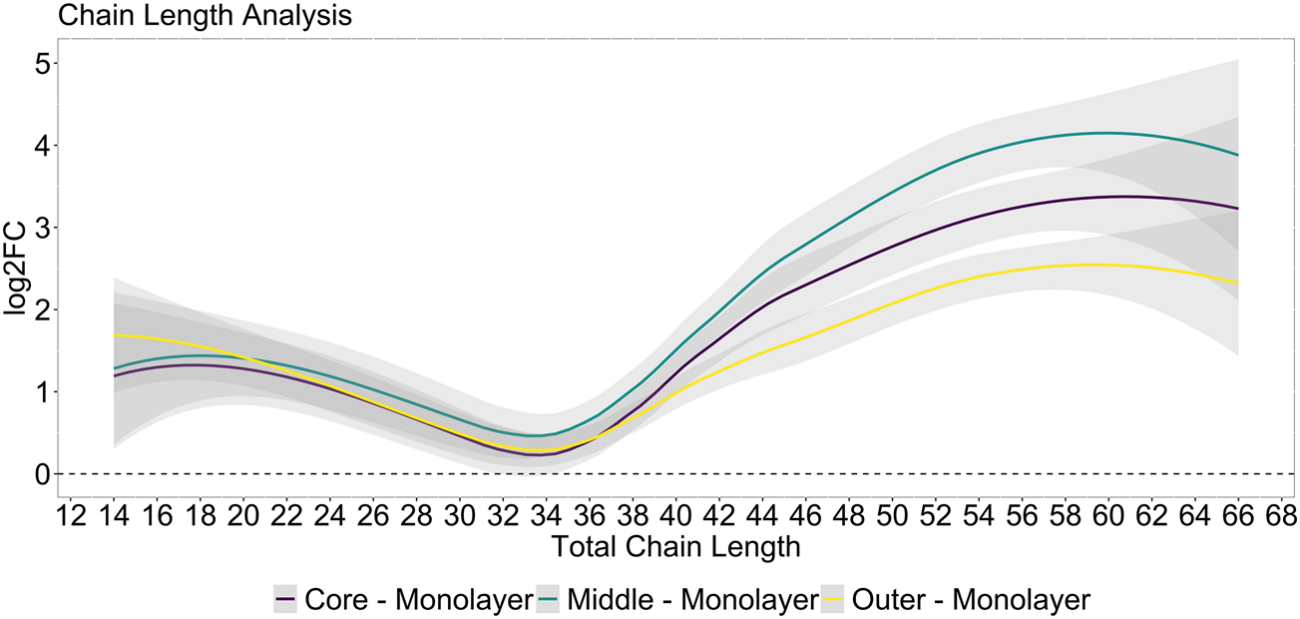
Global lipid chain length analysis as a function of log_2_ fold change ratio between spheroid layer to monolayer culture. Longer chain lengths (total carbon number of 34) are higher in abundance at middle and core layers relative to (2D) monolayer cultures.

We next evaluated the total lipid chain length of the fatty acyl moieties identified across samples. As shown in Figure 5, shorter chain lengths (less than 34 carbons) are elevated at the same levels across the spheroid regions relative to 2D cultures. At the same time, higher production of longer chain lengths appears to be more prominent in the spheroid culture, where the most elevated production of longer chain lengths appears to be in middle and core samples. This suggests different fatty acid metabolism pathways are being utilized between 2D and 3D cultures.

## 4 DISCUSSION

The present lipidomics study aims to characterize the lipidomic profile of 3D and 2D cell cultures of the HCT 116 colorectal cancer cell line. 3D cell culture platforms such as spheroids provide a valuable tool for studying the tumor microenvironment in cancer research as they mimic many of the characteristics that tumors exhibit in the body, such as pH and oxygen gradients. It is widely accepted that cancer cells undergo metabolic changes to support their needs to proliferate. Further, it has been increasingly recognized that lipid metabolism is altered in cancers, which includes increased fatty acid production and uptake from the tumor microenvironment.^47^ As discussed in reviews, cancer cells can acquire fatty acids by either (1) de novo synthesis from non‐lipid substrates; (2) lipid droplet lipolysis; and/or (3) lipophagy of glycerophospholipids.^48^, ^49^ Furthermore, the elongation of fatty acids and their degree of unsaturation (double bond formation) has been a key feature observed in cancer cells. It has been discussed how desaturation of fatty acyl chains as well as the ratios between saturated fatty acyls to monounsaturated fatty acyl (SFA : MUFA) and monounsaturated fatty acyls to polyunsaturated fatty acyls (MUFA : PUFA) can contribute to tumor survival.^50^ More specifically, increased proportions of SFA and MUFA can be found in cancer cell lines and tumor samples compared with non‐malignant cells and benign samples.^51^ This supports other findings that cancer cells with lower proportions of PUFA‐associated lipids are less prone to toxic lipid peroxidation, which can drive the induction of ferroptosis, a regulated form of cell death due to excessive lipid peroxidation.

In addition to the altered fatty acyl composition of cancer cells, accumulation of triacylglycerols (TG) and cholesteryl esters in lipid droplets (LDs) are observed in many cancers. In this study, cholesteryl esters were not successfully detected due to in‐source fragmentation at the fatty acyl‐cholesterol ester bond. Canonically, LDs can serve as sources of FAs that could be shuffled to fulfill cellular needs. A less discussed property of LDs is that they can prevent lipotoxicity by providing cells a buffering capacity to store lipids. They achieve this capacity by sequestering free FAs as TGs to prevent them from incorporating with cytotoxic lipids at high levels such as ceramides or acylcarnitines.^52^ Interestingly, this phenomenon has been observed in several metabolic diseases such as obesity, non‐alcoholic fatty liver disease, and cardiovascular disease.^52^ Indeed, the total TG amount present in the spheroid regions are much more elevated than in 2D cultures (Figure 3A). Furthermore, using Oil Red O staining, we observed an accumulation of LDs in the inner regions of HCT 116 spheroids, which correspond to the increased TG accumulation in spheroids relative to the 2D cultures (Figure S11B). Our findings correlated with previous studies using 3D cell culture to study the lipidomic rewiring in several cancers.^23^, ^53^, ^54^ However, using serial trypsinization, we were able to further decipher specific lipidomic alterations within the spheroid. As our LC–MS‐based lipidomics profiling shows, higher TG levels are present in the middle and core regions of spheroids, where a large portion of the TG species contain PUFAs. Interestingly, it has been suggested that PUFAs are preferentially sequestered in LDs as they can be vulnerable to lipid peroxidation, leading to ferroptosis. At the same time, it was recently reported that extracellular low pH conditions (acidosis) can lead to the activation of TGF‐β2 signaling, thus inducing epithelial‐mesenchymal transition (EMT) and PKC‐zeta‐mediated translocation of CD36, which facilitates FA uptake that are stored in LDs as TGs.^53^

Fatty acid availability can come either from exogenous sources or from de novo synthesis by fatty acid synthase (FASN).^47^, ^55^ Aberrant FASN expression is implicated in several cancers, which is known to induce de novo lipogenesis and is involved in cell proliferation, survival, and invasion.^56^ As a result, the ability to synthesize FAs can make cancer cells independent of the limited environmental availability of precursor substrates. Indeed, there has been considerable interest in the development of FASN inhibitors, which include the next‐generation therapeutics such as TVB‐2640 and yields high anti‐tumor potential and limited systematic toxicity in clinical trials. These promising results from early‐phase trials were an improvement from the first‐generation therapeutics such as C75, orlistat, and cerulenin; although showing great promise as anti‐tumor agents, side effects were present as characterized by severe weight loss.^49^

Cancer cells can activate multiple pathways to obtain its metabolic needs. In addition to exogenous FA uptake, a FADS2‐dependent route of producing PUFAs is also activated. Alternatively, SCD‐1, an enzyme involved in converting saturated fatty acids to monounsaturated fatty acids (MUFA) with double bonds at the Δ9 position, has been shown to be overexpressed in cancer.^47^ However, SCD‐1 requires O_2_ to be active, and thus, O_2_ deprivation can inhibit the activity of the enzyme.^57^ In addition, as tumors grow, their need for unsaturated lipids shifts from endogenous production to the uptake from the microenvironment. FADS2 is a key enzyme in the production of polyunsaturated fatty acids and is more highly expressed in several cancers, including colorectal cancers.^58^

In addition to fatty acid dysregulation, the accumulation of lipid droplets in tumors and 3D cell culture was observed, which provides storage for TGs and cholesteryl esters.^23^ In the current study, we observed a class‐wide increase of TGs in all layers of the spheroid. However, we were unable to detect intact cholesteryl esters in this study as the cholesterol moeity appeared to have been cleaved off, perhaps during the electrospray process. This potential cleavage was gleaned from the data when we generated the extracted ion chromatogram for *m/z* 369.3516, the [M‐H_2_O + H]^+^ form of cholesterol. In this chromatogram, seven peaks are detected eluting in the high organic region of the chromatogram (Figure S12). The greatest increase of TGs was observed in the middle layer and core layer samples, representing the quiescent and necrotic regions of spheroids, respectively, and which have lower oxygen availability. Interestingly, the most abundant TG lipids found in spheroids consisted of a high degree of unsaturation in their FAs (Figure 3). It has been observed that there is a connection between SCD‐dependent or FADS‐dependent unsaturated fatty acid production. Vriens et al. observed that some cancer cells rely on their metabolic plasticity to produce unsaturated lipids.^59^ Additionally, high PUFA abundance has been central to the activation of ferroptosis, which is a non‐apoptotic, iron‐dependent, oxidative cell death process. As recently discussed in a review by Hoy et al., PUFA‐containing lipids are more susceptible to oxidation. We speculate that FADS‐dependent fatty acid metabolism is likely occurring due to the lower oxygen availability in the quiescent and necrotic regions. This hypothesis is supported by the fact that a higher production of TGs composed of PUFAs is observed in spheroids relative to 2D cultures. Finally, total chain length analysis (Figure 5) suggests that longer fatty acyls are also present in spheroids, relative 2D cultures. This comparison further supports that TGs, which are composed of three fatty acyl chains, are much more abundant in spheroids than in 2D cultures. Further investigation is warranted to elucidate the higher levels of PUFA‐containing TGs in the middle and core regions of spheroids.

The glycerophospholipid profile indicated a diverse alteration among the lipid classes. PC lipids and their ether‐linked forms reveal different alterations. For example, PC lipids (Figure S2) with higher degree of unsaturation are more abundant in spheroids than in 2D cultures. Meanwhile, ether‐linked PC (PC‐O) lipids consisting of a saturated fatty acid or MUFA are more abundant in 2D cultures than in spheroids. PE lipids, which are also comprised for ether‐linked (PE‐O) and plasmanyl (PE‐P) lipids, exhibited mild elevation in abundance (Figure S3 and supporting information). Meanwhile, phosphatidylinositol (PI) lipids exhibit species‐specific alterations between spheroids and 2D cultures. PI lipids can be phosphorylated to form phosphatidylinositol monophosphates (PIP), phosphatidylinositol bisphosphates (PIP_2_), or phosphatidylinositol trisphosphates (PIP_3_). These phosphorylated PIs are involved in numerous cell signaling pathways such as membrane trafficking, modulation of P53 activity, and activation of the oncogenic AKT pathway.^49^, ^60^ It is unclear whether the PI lipid levels in spheroids provide indication of altered cell signaling pathways.

Sphingolipids play an important role in cell signaling to control tumor progression and survival. Among the different types of sphingolipids, we detected lipids under the ceramide and neutral glycosphingolipid classes, as well as sphingomyelin and sulfatide. Ceramide is the central lipid of all sphingolipids as it can be further metabolized or transformed by various enzymes.^61^ The accumulation/synthesis of ceramide in response to cellular stress is well‐documented and is known to induce apoptosis, necroptosis, and endoplasmic reticulum (ER) stress.^11^, ^62^, ^63^ Interestingly, ceramide accumulation is observed to occur as a secondary effect after radiation treatment and chemotherapy treatment. In other diseases, ceramide and other sphingolipids have been associated with neuronal cell death, which was previously observed in a mouse model of Niemann–Pick disease, type C (NPC).^64^ It is worth noting that individual ceramide species generated exert different functions in the cell. For example, long chain ceramides containing fatty acyls 16:0, 18:0, and 20:0 are known to be antiproliferative, whereas ceramides containing very long fatty acyls 24:0 or 24:1 promote cellular proliferation.^65^ These ceramides are associated to specific ceramide synthases (CERS), which have propensities toward particular acyl‐CoA to bond with sphingosine.^63^, ^66^ From our current data, it seems to indicate higher levels of sphingolipids in all regions of the spheroid, with the middle region having the highest abundance of all four subclasses (Figure 3B). This could indicate higher levels of ceramide‐mediated cell death is occurring, but further studies are warranted to further investigate these alterations.

Higher production of acylcarnitines were observed in all three spheroid layers. Acylcarnitines are intermediate forms of free FAs that are destined for the mitochondria, where it is further metabolized to acyl‐CoA and carnitine by CPT2. Furthermore, the acyl‐CoA is additionally involved in a multistep process known as β‐oxidation, which ultimately feeds into the Krebs cycle.^47^

## 5 CONCLUSION

In conclusion, this lipidomic profiling revealed several lipid class‐wide alterations and lipid‐species specific changes in HCT 116 spheroids. Lipids associated with lipid droplets such as TGs indicate spatial‐specific elevations in abundance. At the same time, highly unsaturated fatty acyl chains make up most of these TG lipids and are found in the highest abundance in the hypoxic and acidic regions of spheroids. This finding is supported by previous studies by which PUFAs are suggested to be sequestered by cancer cells in LDs, ultimately are prone to peroxidation in the acidic tumor environment leading to ferroptosis‐mediated anticancer effects. Finally, elevated sphingolipid and acylcarnitine production in spheroids suggest apoptosis and β‐oxidation of fatty acids are occurring. The lipidomic signature for each region and cell culture type highlights the importance of understanding the spatial aspects of cancer biology. These results provide additional lipid biomarkers in the tumor microenvironment that can be further studied during therapeutic studies to target pivotal lipid production pathways.

## CONFLICTS OF INTEREST

The authors declare no competing interests.

## AUTHOR CONTRIBUTIONS

FT and ABH designed the experiments. FT completed the experiments and analyzed the data. FT and ABH wrote the manuscript.

## Supporting information


**Table S1:** Internal Standard calculation using EquiSPLASH LIPIDOMIX
**Table S2:** MS‐DIAL Parameter Settings
**Figure S1:** Growth Curve of HCT 116 spheroids
**Figure S2:** Extracted Ion Chromatograms (EIC) of internal standards
**Figure S3:** (PC) abundance profile in HCT 116 spheroids and retention time behavior of each identified lipid
**Figure S4:** (PE) abundance profile in HCT 116 spheroids and retention time behavior of each identified lipid.
**Figure S5:** (PI) abundance profile in HCT 116 spheroids.
**Figure S6:** (PS) abundance profile in HCT 116 spheroids and retention time behavior of PS lipids in reversed‐phase chromatography
**Figure S7:** Cer, HexCer and sphingomyelin abundance profile in HCT 116 spheroids.
**Figure S8:** Acylcarnitine abundance profile in HCT 116 spheroids.
**Figure S9:** Free fatty acid (FA) abundance profile in HCT 116 spheroids.
**Figure S10:** Retention time behavior for free fatty acyls and acylcarnatine lipid species
**Figure S11:** Oil‐Red‐O staining of HCT 116 spheroid cyrosections
**Figure S12:** Extracted ion chromatogram of cholesterol as *m/z* 369.3516.
**Table S3:** Supporting Information File. Microsoft Excel table (.xlsx file) of the curated data, and subclass tables with the normalized molar abundances of each lipid species.

## Data Availability

Raw data are available in the Metabolomics Workbench.
