# Supplementary material for: Lipidomic comparison of 2D and 3D colon cancer cell culture models
Source: J Mass Spectrom. 2022 Aug 26;57(8):e4880. doi: 10.1002/jms.4880 (PMC9526240; doi:10.1002/jms.4880)
Supplement: Supplementary file 1 — Table S1: Internal Standard calculation using EquiSPLASH LIPIDOMIX Table S2: MS‐DIAL Parameter Settings Figure S1: Growth Curve of HCT 116 spheroids Figure S2: Extracted Ion Chromatograms (EIC) of internal standards Figure S3: (PC) abundance profile in HCT 116 spheroids and retention time behavior of each identified lipid Figure S4: (PE) abundance profile in HCT 116 spheroids and retention time behavior of each identified lipid. Figure S5: (PI) abundance profile in HCT 116 spheroids. Figure S6: (PS) abundance profile in HCT 116 spheroids and retention time behavior of PS lipids in reversed‐phase chromatography Figure S7: Cer, HexCer and sphingomyelin abundance profile in HCT 116 spheroids. Figure S8: Acylcarnitine abundance profile in HCT 116 spheroids. Figure S9: Free fatty acid (FA) abundance profile in HCT 116 spheroids. Figure S10: Retention time behavior for free fatty acyls and acylcarnatine lipid species Figure S11: Oil‐Red‐O staining of HCT 116 spheroid cyrosections Figure S12: Extracted ion chromatogram of cholesterol as m/z 369.3516. Table S3: Supporting Information File. Microsoft Excel table (.xlsx file) of the curated data, and subclass tables with the normalized molar abundances of each lipid species. [file JMS-57-e4880-s001.docx]

**Supporting Information for:**

**Lipidomic Comparison of 2D and 3D Colon Cancer Cell Culture Models**

Fernando Tobias^1^ and Amanda B. Hummon^1,2^*

1. Department of Chemistry and Biochemistry, The Ohio State University, Columbus, OH 43210
2. Comprehensive Cancer Center, The Ohio State University, Columbus, OH 43210

Corresponding author: Amanda B. Hummon

Email: [hummon.1@osu.edu](mailto:hummon.1@osu.edu)

**Table of Contents**

Supporting Table 1: Internal Standard calculation using EquiSPLASH LIPIDOMIX

Supporting Table 2: MS-DIAL Parameter Settings

Supporting Figure 1: Growth Curve of HCT 116 spheroids

Supporting Figure 2: Extracted Ion Chromatograms (EIC) of internal standards

Supporting Figure 3: (PC) abundance profile in HCT 116 spheroids and retention time behavior of each identified lipid

Supporting Figure 4: (PE) abundance profile in HCT 116 spheroids and retention time behavior of each identified lipid.

Supporting Figure 5: (PI) abundance profile in HCT 116 spheroids.

Supporting Figure 6: (PS) abundance profile in HCT 116 spheroids and retention time behavior of PS lipids in reversed-phase chromatography

Supporting Figure 7: Cer, HexCer and sphingomyelin abundance profile in HCT 116 spheroids.

Supporting Figure 8: Acylcarnitine abundance profile in HCT 116 spheroids.

Supporting Figure 9: Free fatty acid (FA) abundance profile in HCT 116 spheroids.

Supporting Figure 10: Retention time behavior for free fatty acyls and acylcarnatine lipid species

Supporting Figure 11: Oil-Red-O staining of HCT 116 spheroid cyrosections

Supporting Figure 12: Extracted ion chromatogram of cholesterol as *m/z* 369.3516.

Supporting Table 3: Supporting Information File. Microsoft Excel table (.xlsx file) of the curated data, and subclass tables with the normalized molar abundances of each lipid species.

| Resuspension volume (μL) | 100 |  |  |  |
| --- | --- | --- | --- | --- |
| Protein content (μg) | 200 |  |  |  |
|  | Negative Mode |  |  |  |
|  | injection volume (μL) | IS Volume (μL) | Stock Conc. (μM) | **pmol/μg protein** |
| PC | 5 | 10 | 133 | **0.333** |
| LPC | 5 | 10 | 189 | **0.473** |
| PE | 5 | 10 | 141 | **0.353** |
| LPE | 5 | 10 | 205 | **0.513** |
| PG | 5 | 10 | 131 | **0.328** |
| PI | 5 | 10 | 118 | **0.295** |
| PS | 5 | 10 | 129 | **0.323** |
| TAG | 5 | 10 | 123 | **0.308** |
| DAG | 5 | 10 | 170 | **0.425** |
| MAG | 5 | 10 | 275 | **0.688** |
| CholEster | 5 | 10 | 152 | **0.380** |
| SM | 5 | 10 | 135 | **0.338** |
| Cer | 5 | 10 | 188 | **0.470** |
|  | Positive mode |  |  |  |
|  | injection volume (μL) | IS Volume (μL) | Stock Conc. (μM) | **pmol/μg protein** |
| PC | 2 | 10 | 133 | **0.133** |
| LPC | 2 | 10 | 189 | **0.189** |
| PE | 2 | 10 | 141 | **0.141** |
| LPE | 2 | 10 | 205 | **0.205** |
| PG | 2 | 10 | 131 | **0.131** |
| PI | 2 | 10 | 118 | **0.118** |
| PS | 2 | 10 | 129 | **0.129** |
| TAG | 2 | 10 | 123 | **0.123** |
| DAG | 2 | 10 | 170 | **0.170** |
| MAG | 2 | 10 | 275 | **0.275** |
| CholEster | 2 | 10 | 152 | **0.152** |
| SM | 2 | 10 | 135 | **0.135** |
| Cer | 2 | 10 | 188 | **0.188** |

**Supporting Table 1:** Internal Standard determination using the equiSPLASH LIPIDOMIX standard. 10 μL of equiSPLASH was added to each sample lysate prior to lipid extraction. Prior to MS analysis, the samples were resuspended in 100 μL of solvent, where 5 μL was injected for negative mode analysis and 2 μL was injected for positive mode analysis. The resulting normalized concentration (bolded) was then inputted in MS-DIAL to quantify the identified lipids.

| Data Collection Tab | Positive mode | Negative mode |
| --- | --- | --- |
| Mass accuracy |  |  |
| MS1 tolerance | 0.01 | 0.01 |
| MS2 tolerance | 0.025 | 0.025 |
|  |  |  |
| Peak detection Tab |  |  |
| Minimum peak height | 1000 | 1000 |
| Mass slice width | 0.1 | 0.1 |
|  |  |  |
| Identification Tab |  |  |
| Solvent type | Ammonium formate | Ammonium formate |
| Retention time tolerance | 100 | 100 |
| Accurate mass tolerance (MS1) | 0.01 | 0.01 |
| Accurate mass tolerance (MS2) | 0.05 | 0.05 |
| Identification score cut off | 80% | 80% |
| Use retention time for scoring | Unchecked | Unchecked |
| Use retention time for filtering | Unchecked | Unchecked |
|  |  |  |
| Adduct Tab |  |  |
| Molecular species | [M+H]+,[M+NH4+]+,  [M+Na]+,  [M-H2O+H]+ | [M-H]-,[M-H2O-H]-,[M+Cl]-,  [M-FA-H]-,[M+Hac-H]-,  [2M-H]-,[M-2H]2- |
|  |  |  |
| Alignment Tab |  |  |
| Retention time tolerance | 0.05 | 0.08 |
| MS1 tolerance | 0.015 | 0.015 |

**Supporting Table 2:** MS-DIAL Analysis parameter settings for positive and negative ion modes. Version 4.48 was used.

**
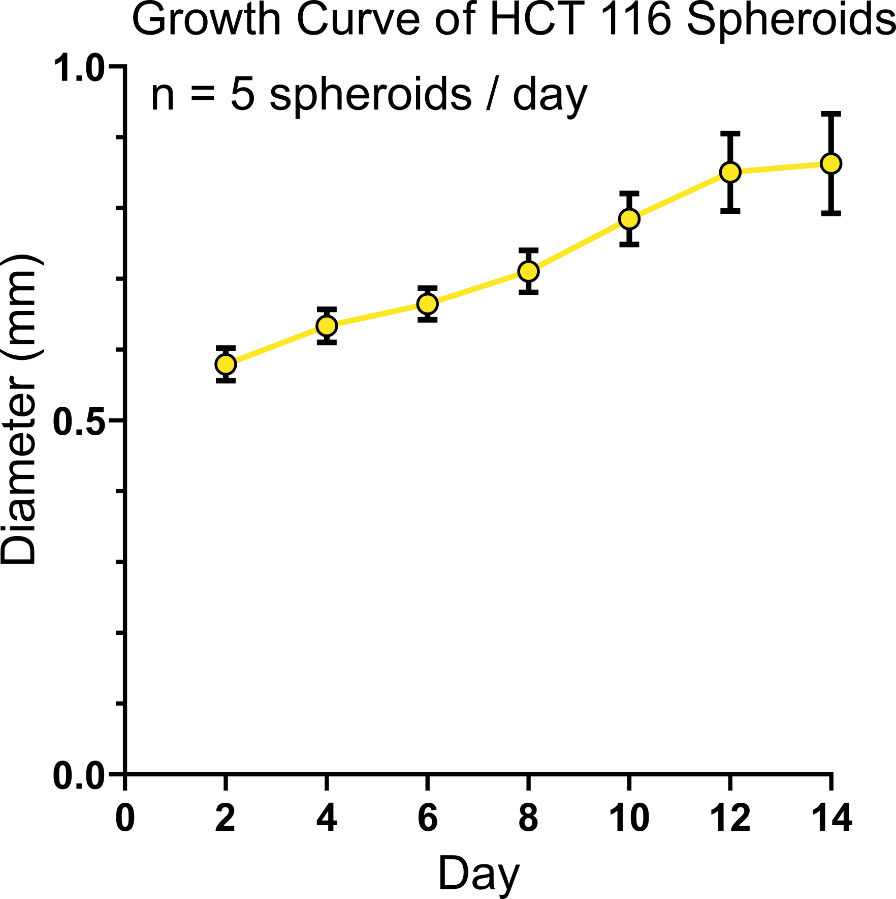
**

**Supporting Figure 1**: Growth curve of HCT 116 spheroids during the 14-day culturing process. The same five spheroids were measured with a cellular phone camera attached to a microscope. Resulting photographs were uploaded to imageJ where there were measured to obtain averaged spheroid diameters. Error bars represent the standard deviation.


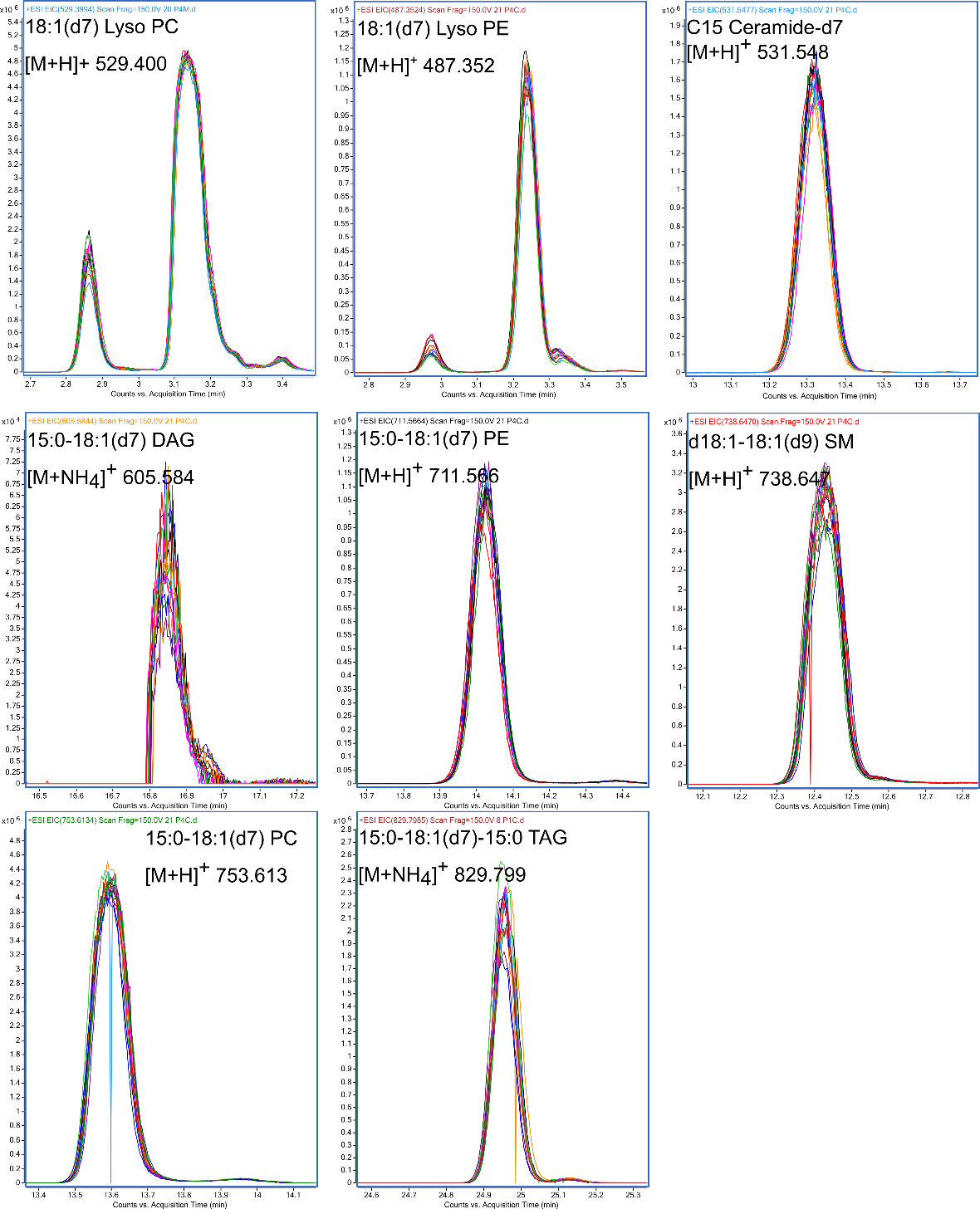


**Supporting Figure 2:** Extracted ion chromatogram of EquiSPLASH internal standards showing consistent retention time and abundance across samples. The corresponding mass-to-charge values were used to generate the chromatograms from the biological samples throughout the study.


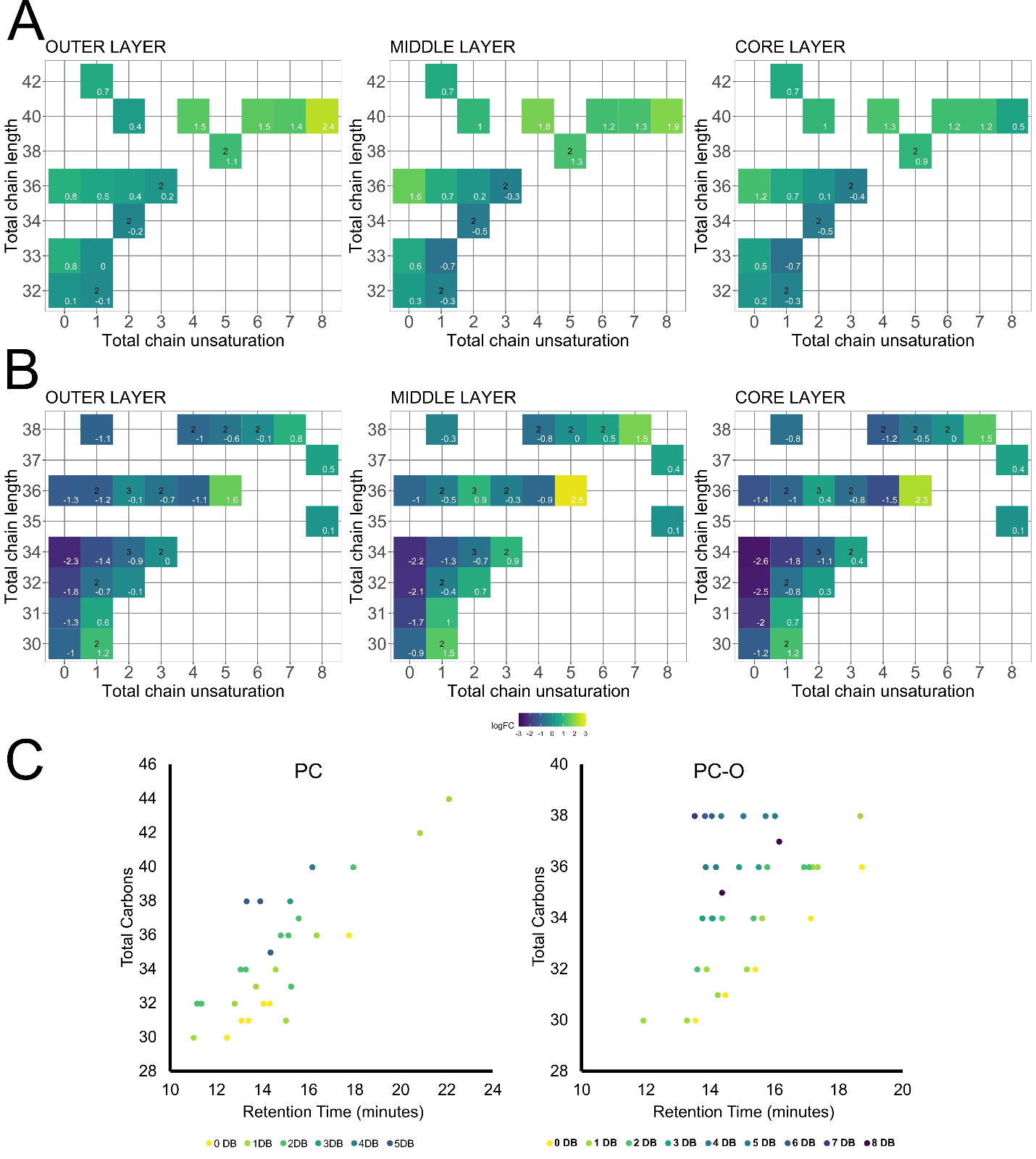


**Supporting Figure 3**: **Phosphatidylcholine (PC) abundance profile in HCT 116 spheroids and retention time behavior of each identified lipid.** A: PC lipids, B: Ether-linked PC-O lipids. The numerical values inside each pixel correspond to the fold-change ratio (spheroid layer / 2D monolayer) of that lipid species. C: Retention time behavior for phosphatidylcholine and sphingomyelin lipid species. 0 DB through 8 DB represent the cumulative number of double bonds in the fatty acyl chains, while the total carbon represents the cumulative number of carbons in the fatty acyl chains.


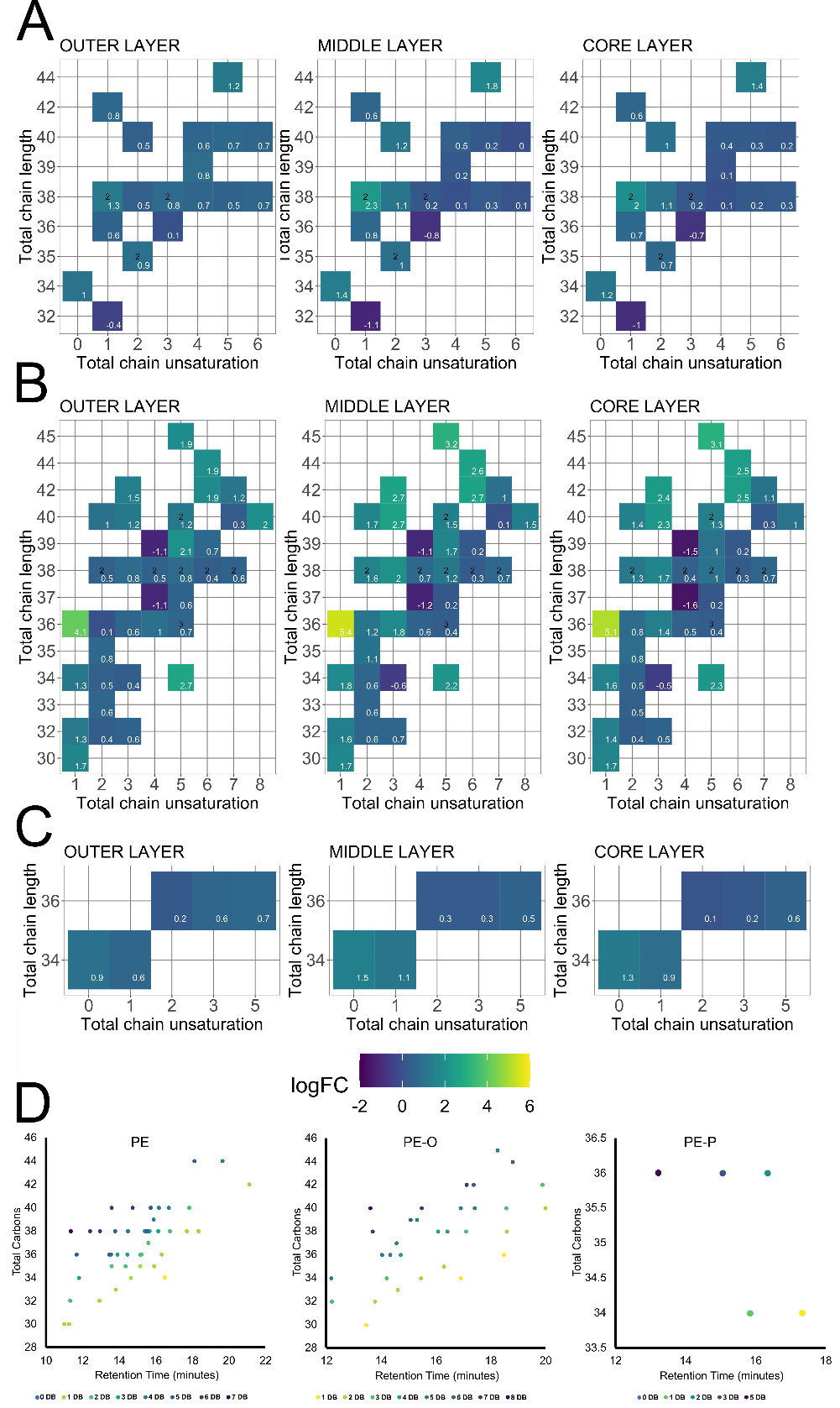


**Supporting Figure 4: Phosphatidylethanolamine (PE) abundance profile in HCT 116 spheroids and retention time behavior of each identified lipid.** A: PE lipids, B: ether-linked PE-O lipids, C: ether-linked PE-P lipids. The numerical values inside each pixel correspond to the fold-change ratio (spheroid layer / 2D monolayer) of that lipid species. D: 0 DB through 8 DB represent the cumulative number of double bonds in the fatty acyl chains, while the total carbon represents the cumulative number of carbons in the fatty acyl chains.

**
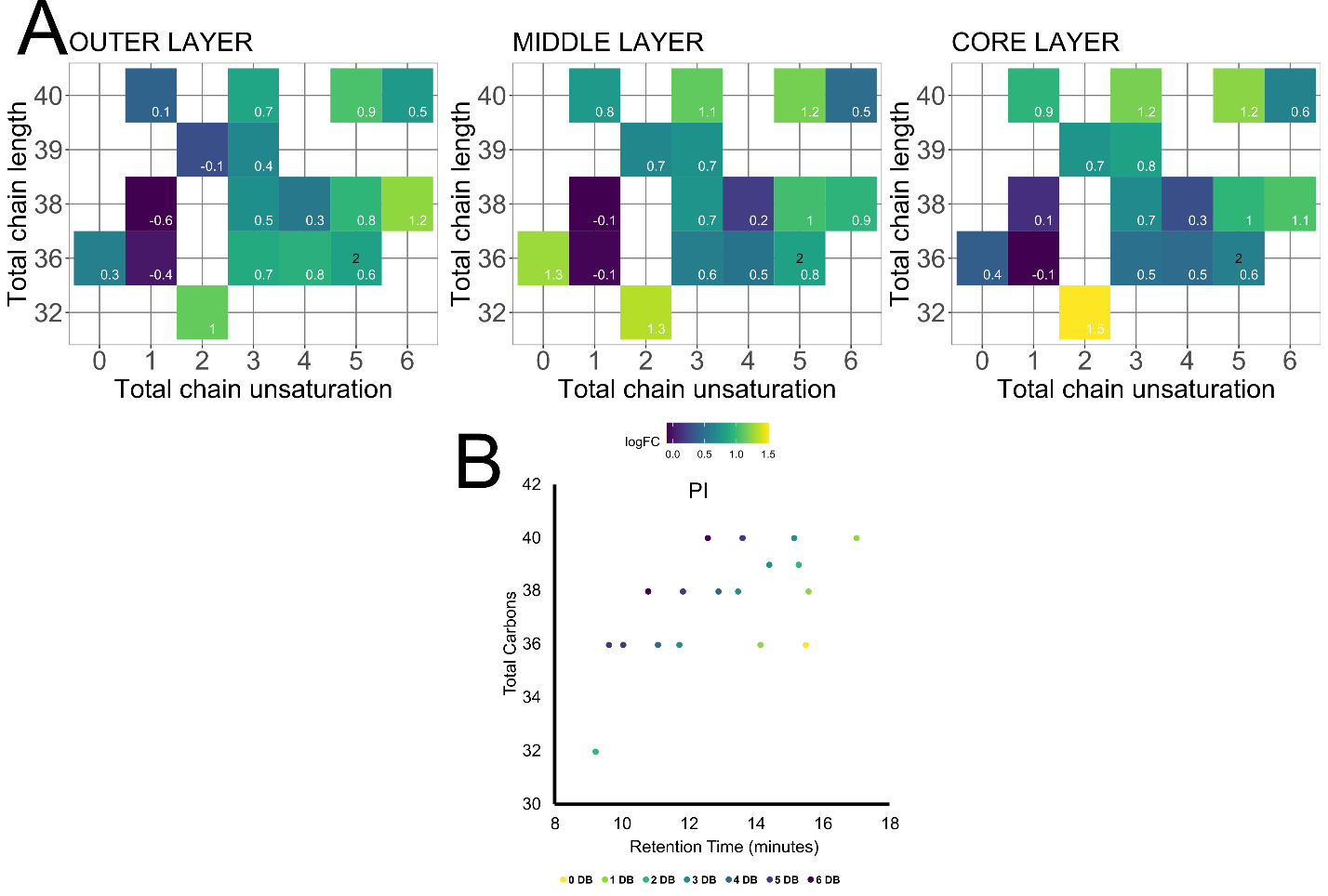
**

**Supporting Figure 5:** **Phosphatidylinositol (PI) abundance profile in HCT 116 spheroids.** A: The numerical values inside each pixel correspond to the fold-change ratio (spheroid layer / 2D monolayer) of that lipid species. B: Retention time behavior of all PI species detected and identified by MS/MS in the study. 0 DB through 6 DB represent the cumulative number of double bonds in the fatty acyl chains, while the total carbon represents the cumulative number of carbons in the fatty acyl chains.


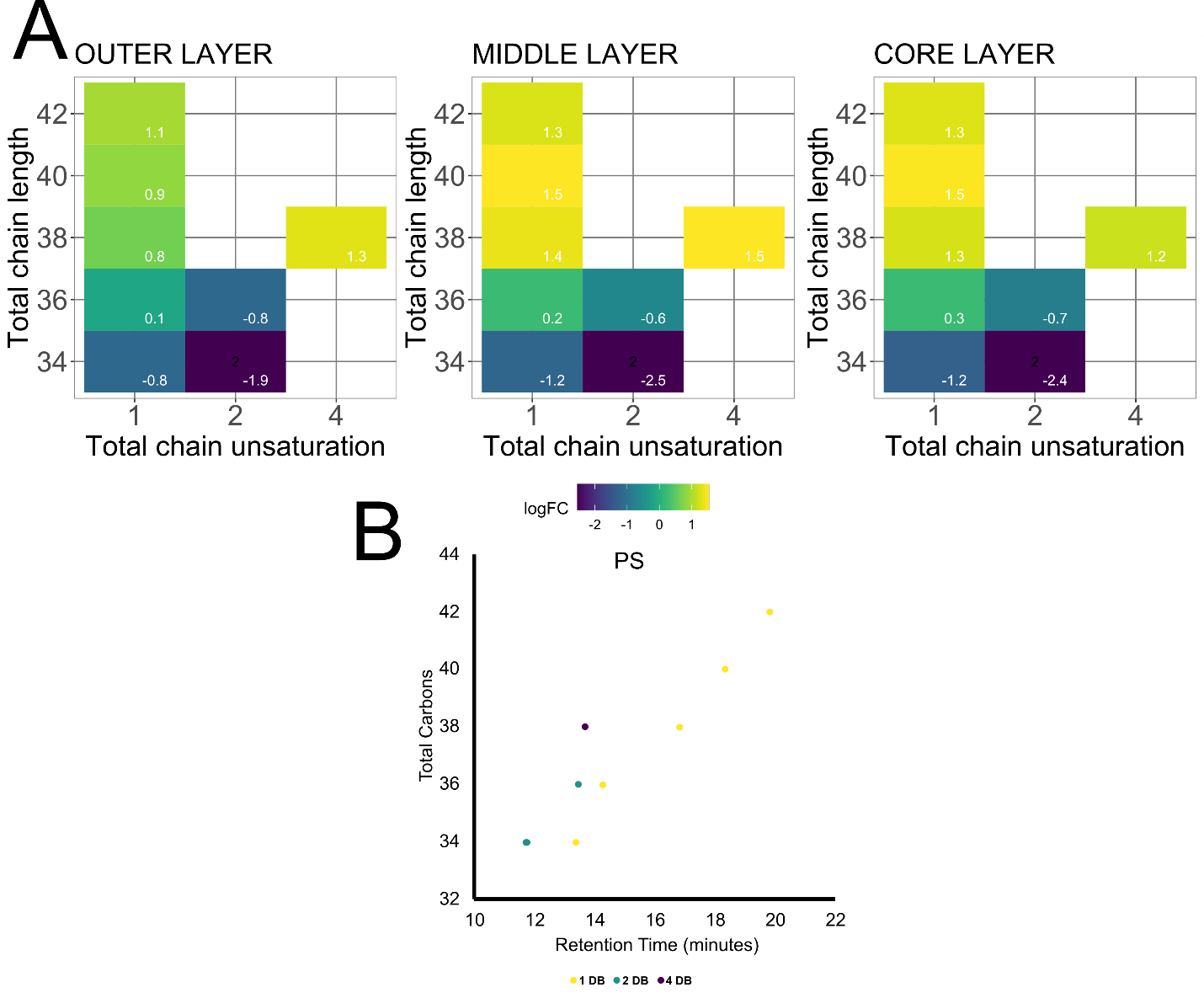


**Supporting Figure 6: Phosphatidyserine (PS) abundance profile in HCT 116 spheroids** **and retention time behavior of PS lipids in reversed-phase chromatography.** A: The numerical values inside each pixel correspond to the fold-change ratio (spheroid layer / 2D monolayer) of that lipid species. B: Retention time behavior of all PS species detected and identified by MS/MS in the study. 1 DB through 4 DB represent the cumulative number of double bonds in the fatty acyl chains, while the total carbon represents the cumulative number of carbons in the fatty acyl chains.


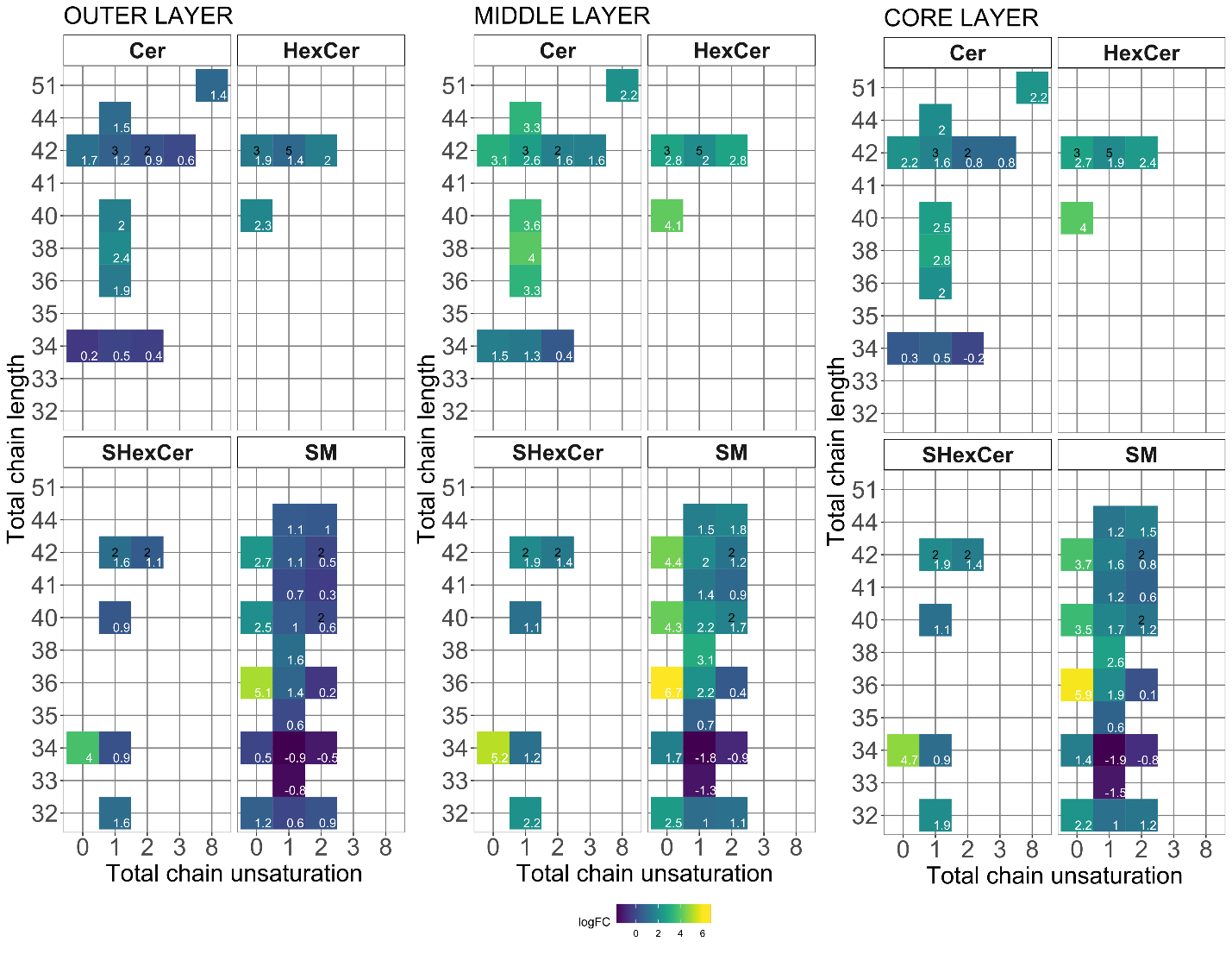


**Supporting Figure 7: Cer, HexCer and sphingomyelin abundance profile in HCT 116 spheroids.** The numerical values inside each pixel correspond to the fold-change ratio (spheroid layer / 2D monolayer) of that lipid species.


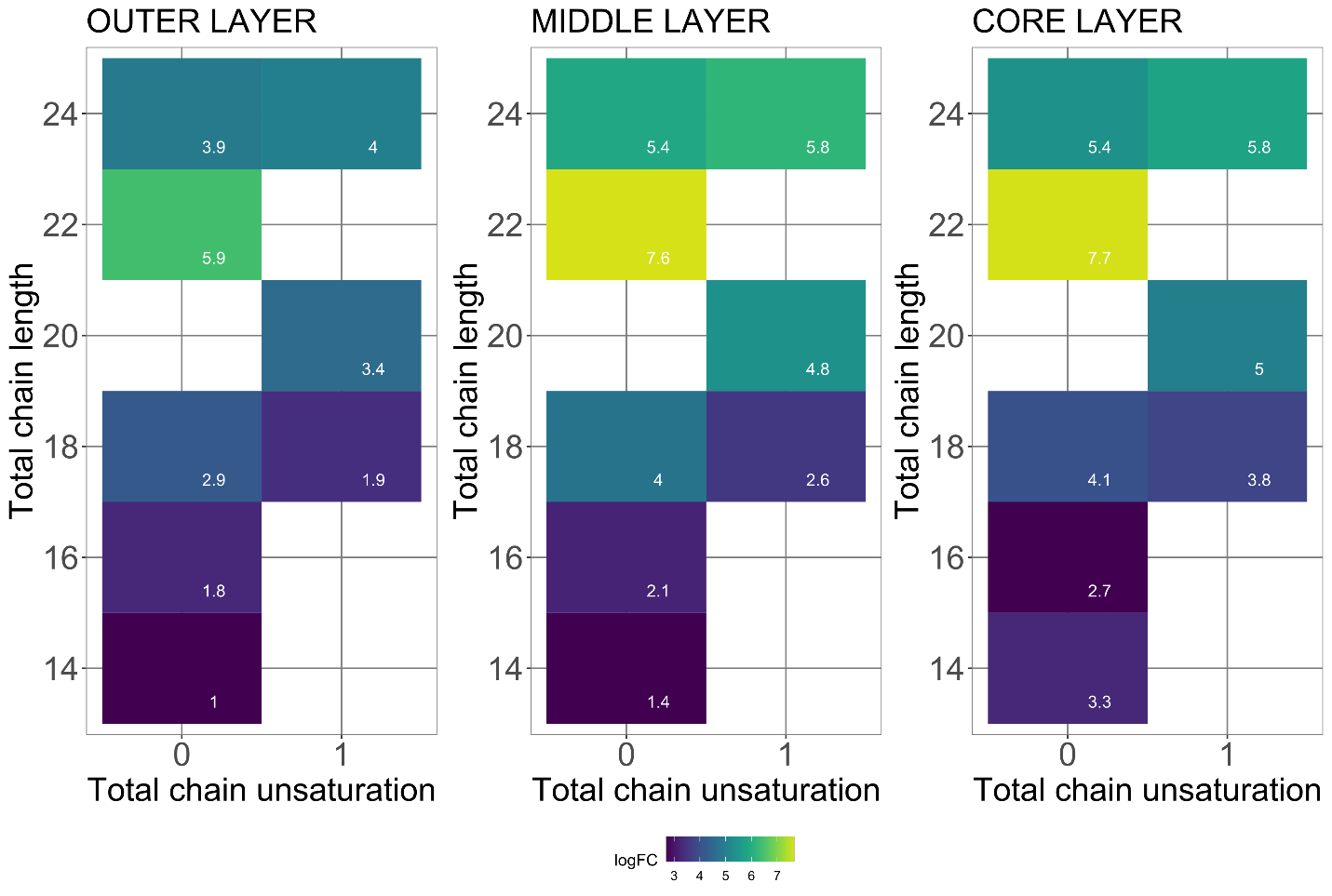


**Supporting Figure 8: Acylcarnitine abundance profile in HCT 116 spheroids.** The numerical values inside each pixel correspond to the fold-change ratio (spheroid layer / 2D monolayer) of that lipid species.

**
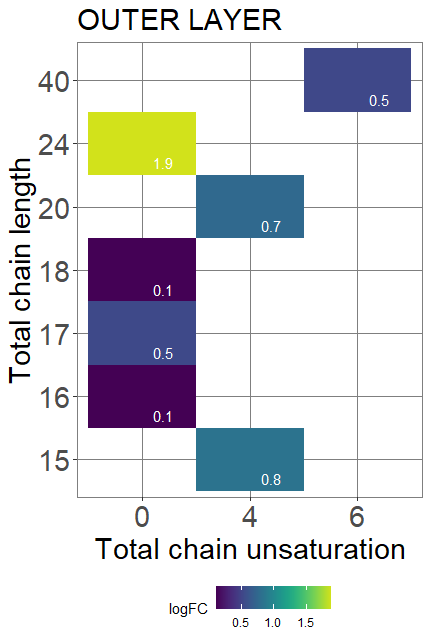

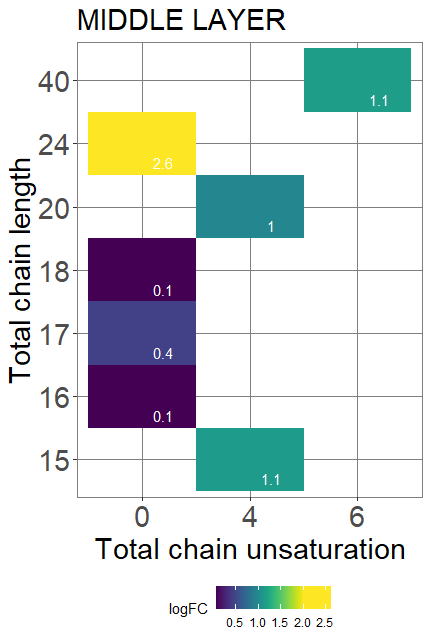

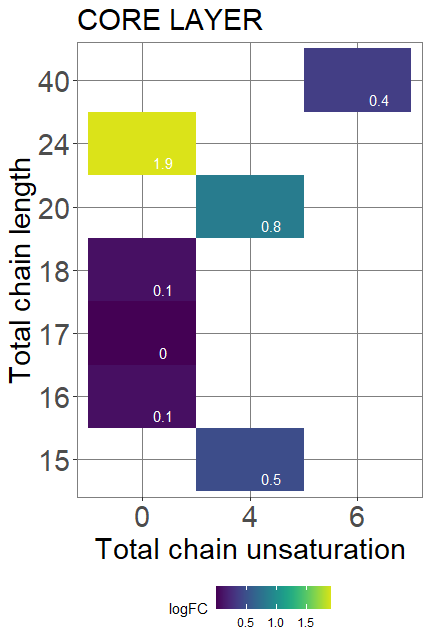
**

**Supporting Figure 9: Free fatty acid (FA) abundance profile in HCT 116 spheroids.** The numerical values inside each pixel correspond to the fold-change ratio (spheroid layer / 2D monolayer) of that lipid species.


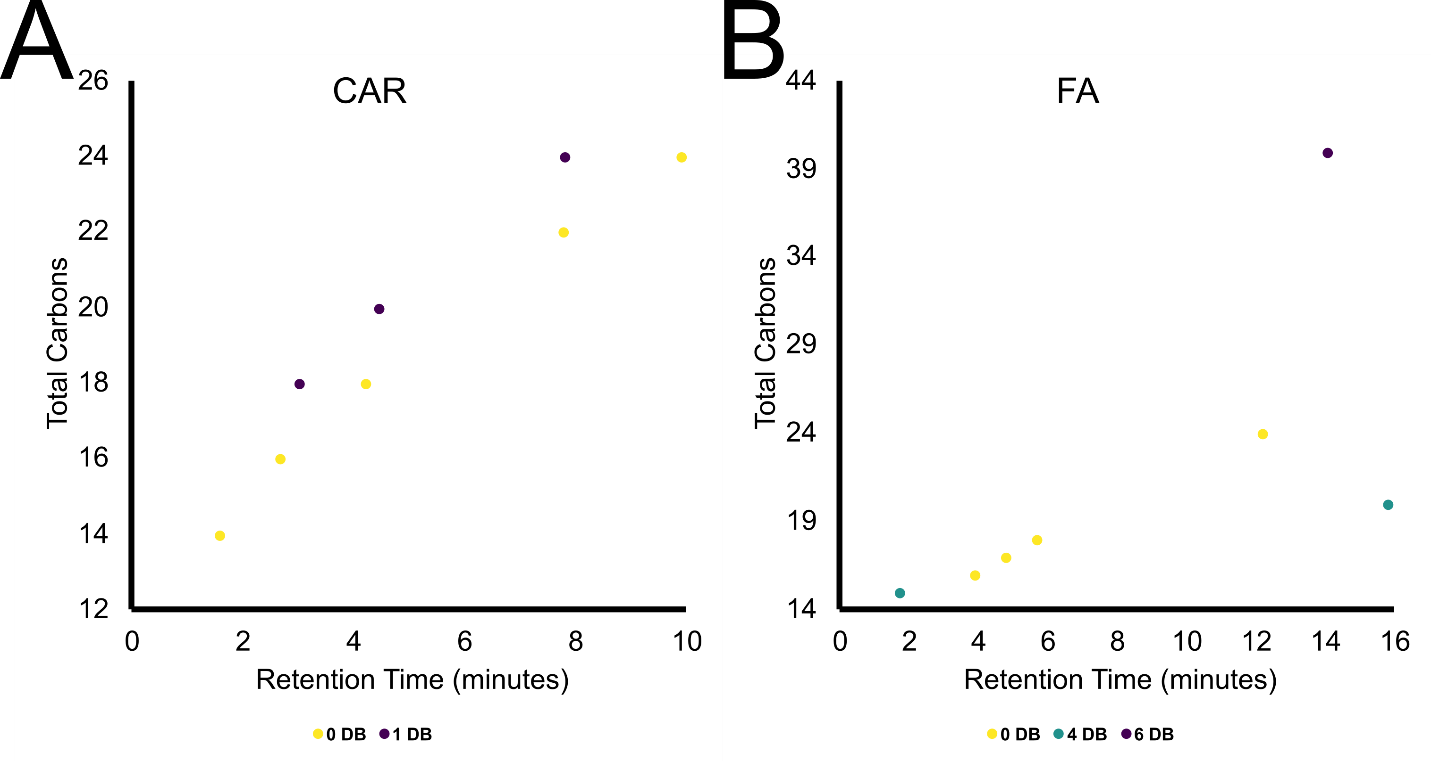


**Supporting Figure 10:** **Retention time behavior for free fatty acids and acylcarnatine lipid species.** A: Acylcarnitine retention time behavior plot B: free fatty acids retention time behavior plot. 0 DB, 1 DB, 4 DB, and 6 DB represent the cumulative number of double bonds in the fatty acyl chains, while the total carbon represents the cumulative number of carbons in the fatty acyl chains.


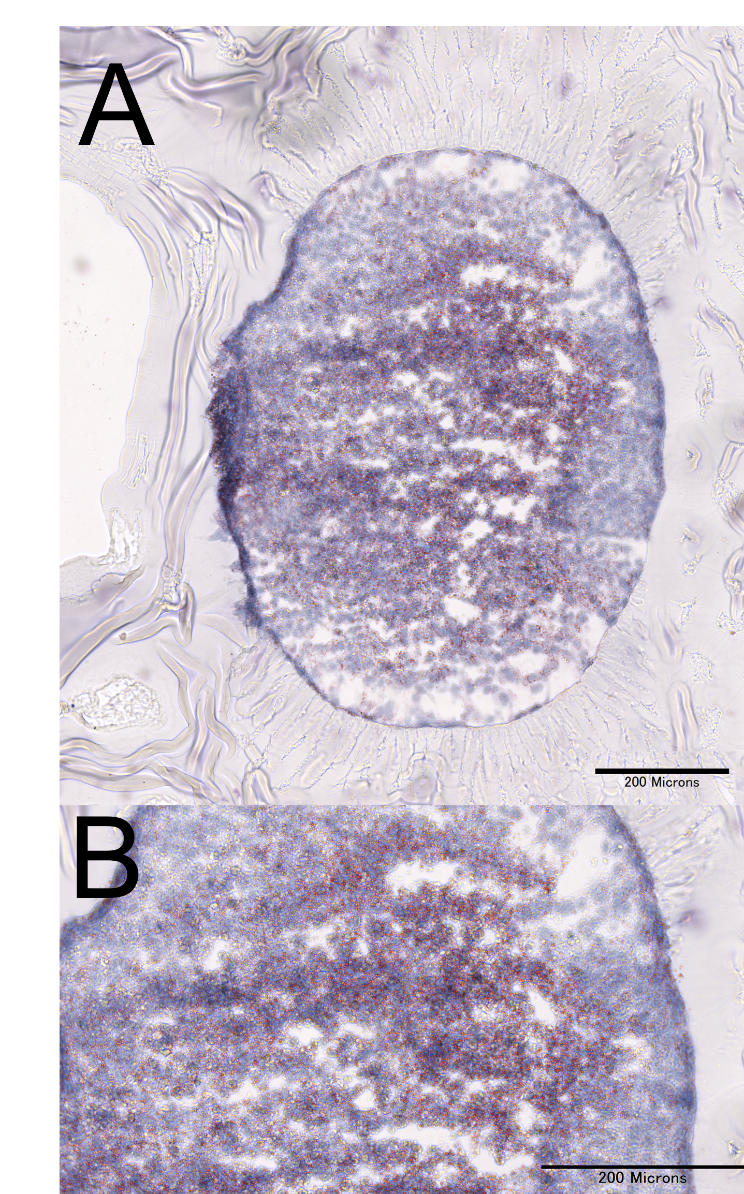


**Supporting Figure 11: Oil-Red-O staining of HCT 116 spheroid sections.** A: Whole-spheroid image of a cryosectioned HCT 116 spheroid at day 14. B: Close-up view of the spheroid, highlighting the lipid droplets in the center region. Scale bar is 200 μm.


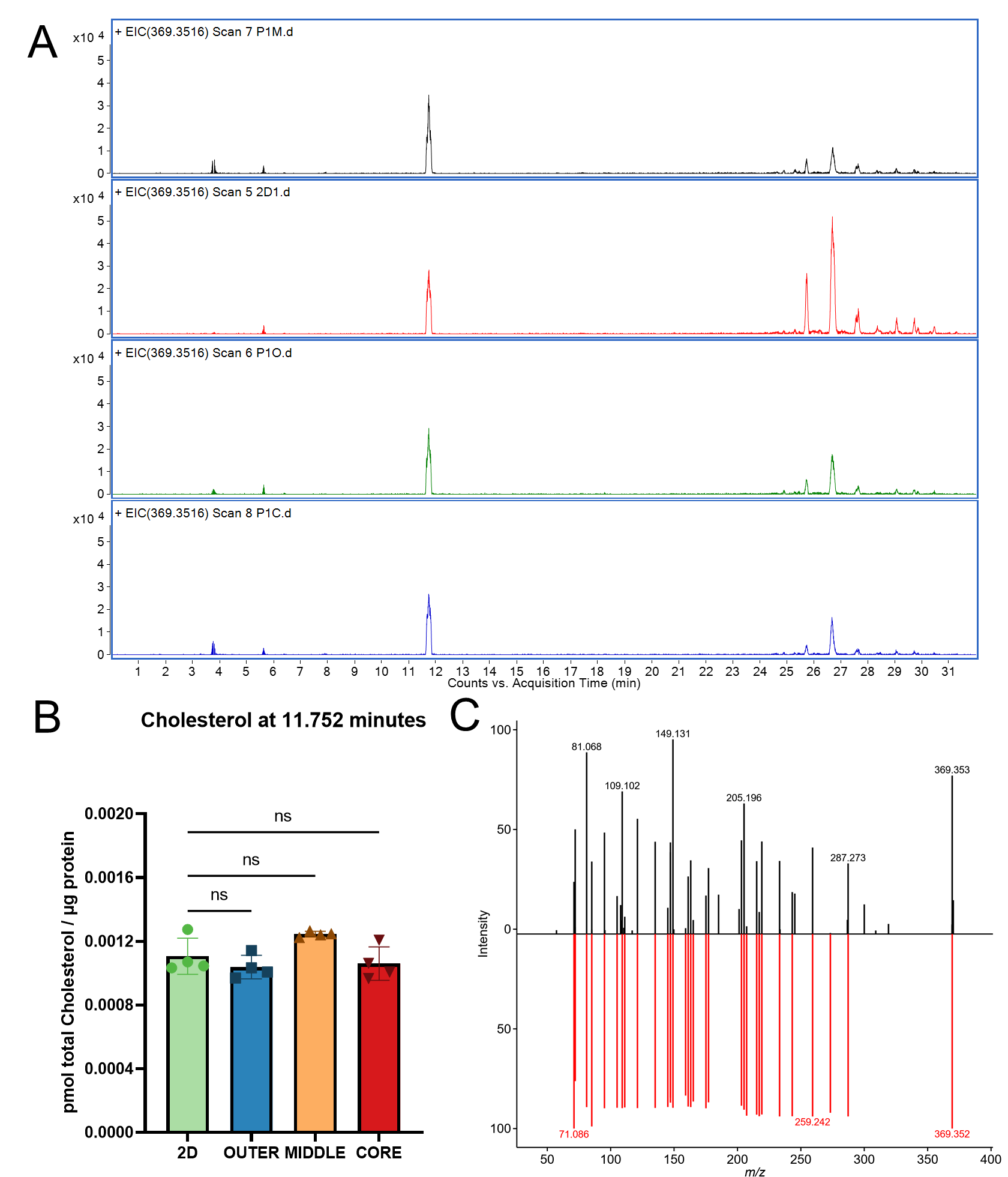


**Supporting Figure 12: Extracted ion chromatogram of cholesterol as *m/z* 369.3516.** A: Representative EICs of *m/z* 369.3516, where free cholesterol can be seen eluting at 11.5 minutes. The peaks at 25 - 30 minutes are cholesterol fragments from cholesteryl esters, B: cholesterol abundance plots, C: experimental (top) versus de novo (bottom) tandem mass spectra from MS-FINDER of *m/z* 369.3516 at 11.5 minutes.
